# Supplementary material for: FcγRIIB controls antibody-mediated target cell depletion by ITIM-independent mechanisms
Source: Cell Rep. 2022 Jul 19;40(3):111099. doi: 10.1016/j.celrep.2022.111099 (PMC9638011; doi:10.1016/j.celrep.2022.111099)
Supplement: Document S1. Figures S1–S6 [file mmc1.pdf]

Cell Reports, Volume *40*

Supplemental information

FcγRIIB controls antibody-mediated target cell depletion by ITIM-independent mechanisms

Alexander P. Simpson, Ali Roghanian, Robert J. Oldham, H.T. Claude Chan, Christine A. Penfold, Hyung J. Kim, Tatyana Inzhelevskaya, C. Ian Mockridge, Kerry L. Cox, Yury D. Bogdanov, Sonya James, Alison L. Tutt, Daniel Rycroft, Peter Morley, Lekh N. Dahal, Ingrid Teige, Björn Frendeus, Stephen A. Beers, and Mark S. Cragg

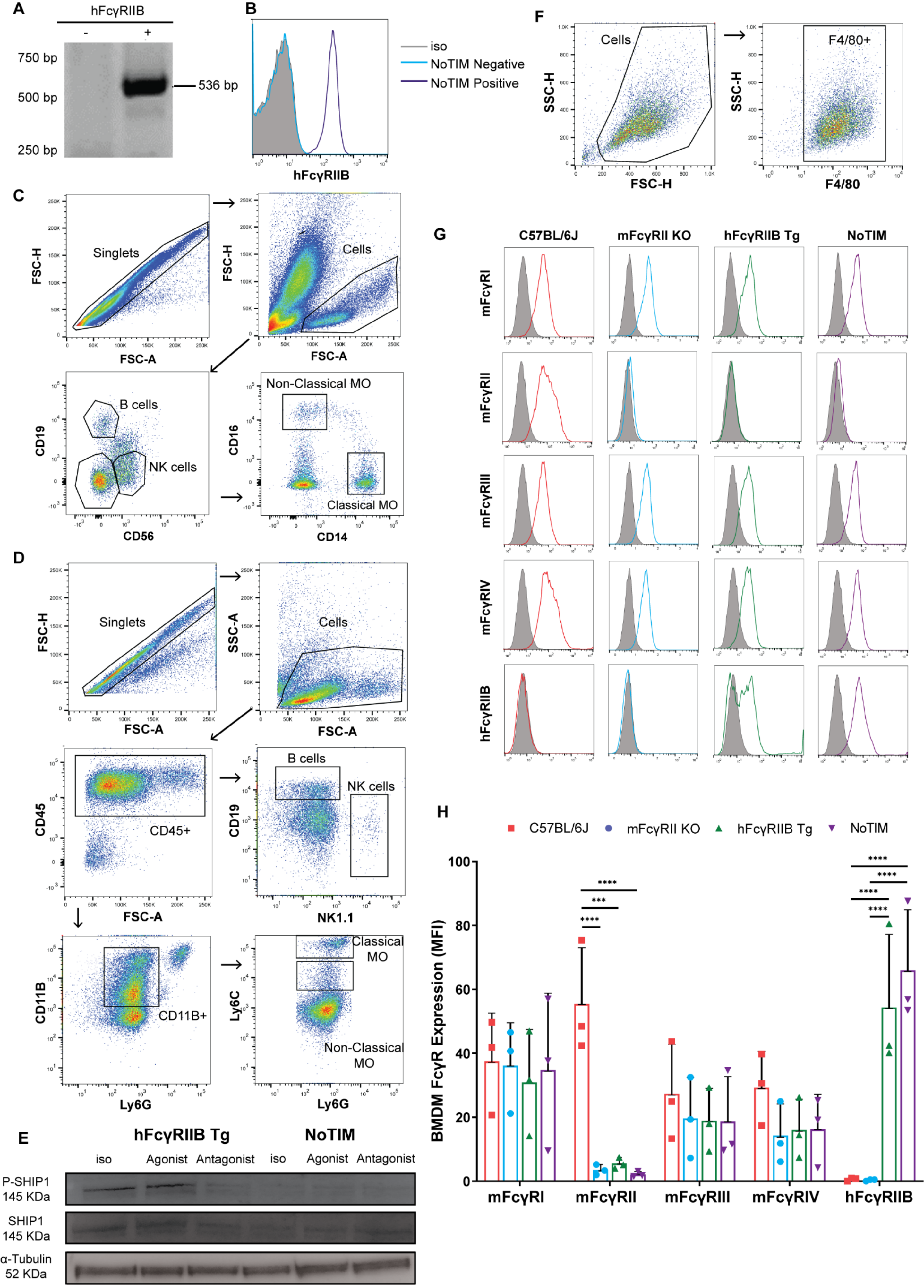
A
hFcγRIIB
-
+
750 bp
500 bp
250 bp
536 bp
B
iso
NoTIM Negative
NoTIM Positive
hFcγRIIB
C
Singlets
FSC-H
FSC-A
Cells
B cells
NK cells
CD19
CD56
Non-Classical MO
Classical MO
CD16
CD14
D
Singlets
Cells
SSC-A
CD45+
CD45
B cells
NK cells
NK1.1
CD11B+
CD11B
Ly6G
Classical MO
Non-Classical MO
Ly6C
E
hFcγRIIB Tg
NoTIM
iso
Agonist
Antagonist
P-SHIP1 145 KDa
SHIP1 145 KDa
α-Tubulin 52 KDa
F
Cells
SSC-H
FSC-H
F4/80+
F4/80
G
C57BL/6J
mFcγRII KO
hFcγRIIB Tg
NoTIM
mFcγRI
mFcγRII
mFcγRIII
mFcγRIV
hFcγRIIB
H
C57BL/6J
mFcγRII KO
hFcγRIIB Tg
NoTIM
BMDM FcγR Expression (MFI)
mFcγRI
mFcγRII
mFcγRIII
mFcγRIV
hFcγRIIB
****
***

FIGURE S1

Figure S1, related to Figure 2. Characterization of the hFcγRIIB NoTIM mouse. A) The presence of the NoTIM transgene was assessed by PCR amplification from genomic DNA using gene-specific primers to identify positive and negative progeny. The transgene included the 400bp region upstream of the human start codon to foster human tissue-specific gene expression. B) flow cytometry was then used to confirm the cell surface expression of the receptor. Grey: isotype; blue: negative sample; purple: NoTIM sample. Representative histogram gated on $B220^{+}CD19^{+}$ cells. C) Flow cytometric gating strategy used to identify human B cells, NK cells, classical and non-classical monocytes (MO) as used in Fig. 2C. D) Flow cytometric gating strategy used to identify murine B cells, NK cells, classical and non-classical monocytes (MO) as used in Fig. 2C. E) Splenic B cells were isolated from NoTIM and hFcγRIIB Tg mice and stimulated with hFcγRIIB specific mAb. Lysates were then probed for phosphorylated SHIP-1 (P-SHIP1), total SHIP-1 and α tubulin as a loading control. F-H) To understand if the loss of the endogenous mFcγRII and the introduction of the hFcγRIIB transgene impacted on the expression of activating mFcγRs, BMDMs were differentiated from the various mouse strains and flow cytometry used to investigate cell surface expression of FcγRs. Expression of FcγR on BMDMs derived from C57BL/6J WT, mFcγRII $^{-/-}$, hFcγRIIB Tg and NoTIM mice is shown. Gating shown in (E) with representative histograms for FcγR shown in (F) and the mean fluorescence intensity (MFI) from 3 independent experiments combined in (G); grey = isotype control. Color represents the relevant FcγR. Columns represent the mean (+SD). Statistical analyses were conducted using a one-way ANOVA with Tukey's multiple comparisons test. * $P \leq 0.05$, ** $P \leq 0.01$, *** $P \leq 0.001$, **** $P \leq 0.0001$.

FIGURE S2

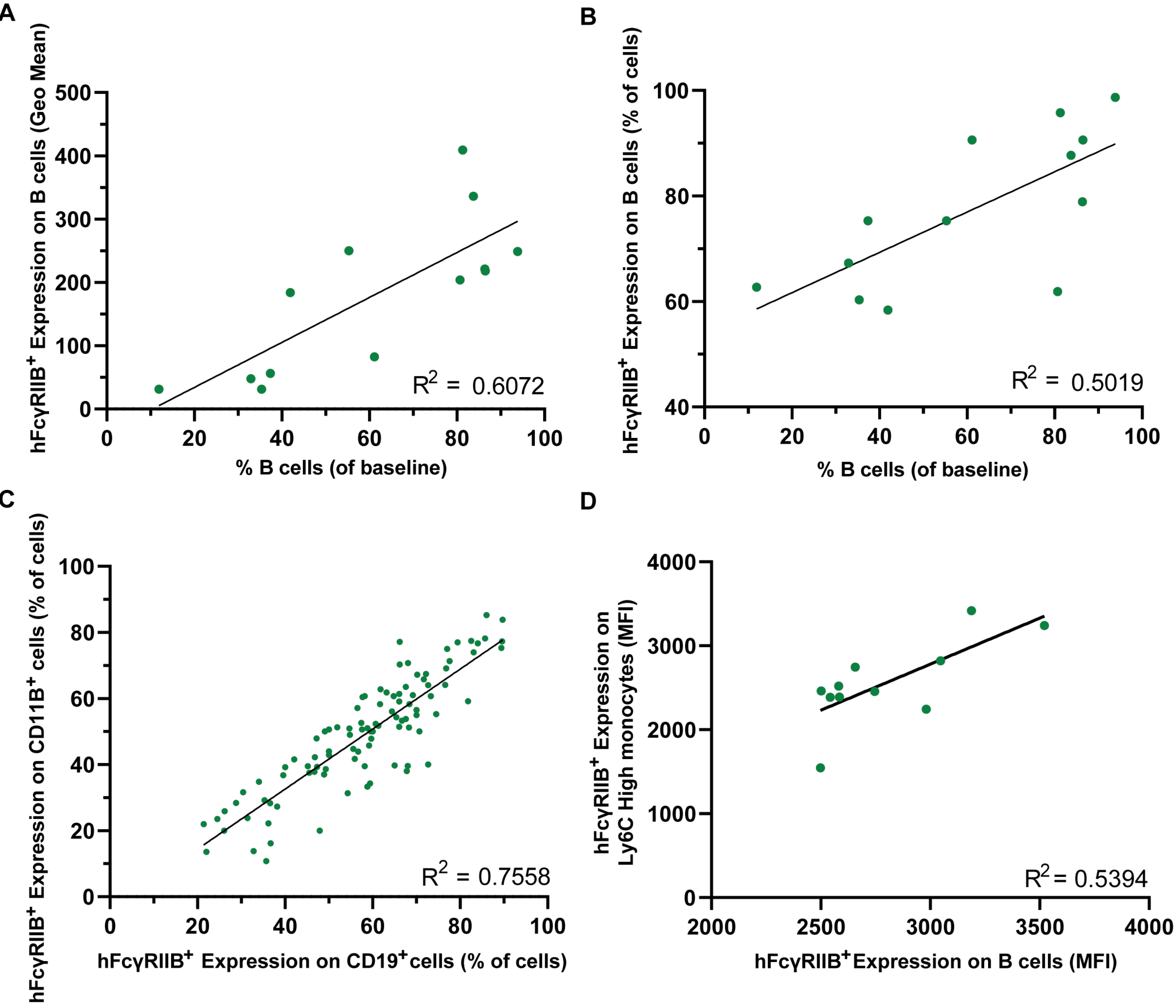

A
hFcγRIIB+ Expression on B cells (Geo Mean)
500
400
300
200
100
0
0
20
40
60
80
100
% B cells (of baseline)
R2 = 0.6072
B
hFcγRIIB+ Expression on B cells (% of cells)
100
80
60
40
0
20
40
60
80
100
% B cells (of baseline)
R2 = 0.5019
C
hFcγRIIB+ Expression on CD11B+ cells (% of cells)
100
80
60
40
20
0
0
20
40
60
80
100
hFcγRIIB+ Expression on CD19+cells (% of cells)
R2 = 0.7558
D
hFcγRIIB+ Expression on Ly6C High monocytes (MFI)
4000
3000
2000
1000
0
2000
2500
3000
3500
4000
hFcγRIIB+Expression on B cells (MFI)
R2 = 0.5394

Figure S2, related to Figure 3. Relationship between hFcγRIIB Tg expression and anti-mCD20 mAb-mediated depletion. A) The expression of hFcγRIIB on B cells from hFcγRIIB Tg mice was measured by flow cytometry prior to the anti-mCD20 (α-mCD20) mAb treatment and correlated to the % remaining B cells (as a % of pre-bleed) on day 14 after treatment from experiments shown in Fig. 4. The R^2 values were calculated for from the line of best fit (R^2 = 0.6072; p 0.0017). B) The % of hFcγRIIB positive B cells from hFcγRIIB Tg mice was measured by flow cytometry prior to α-mCD20 mAb treatment and correlated to the % remaining B cells (as a % of pre-bleed) on day 14 from experiments shown in Fig. 4 (R^2 = 0.5019; p 0.0067). C) Correlation of % positivity of the hFcγRIIB transgene between B cells and monocytes (R^2 0.7558; p <0.0001). Given the tight correlation, and ease of screening the majority of mice were screened solely for hFcγRIIB expression on B cells. D) The MFI of hFcγRIIB on splenic Ly6C high monocytes from healthy hFcγRIIB Tg mice was measured by flow cytometry and correlated with the MFI of hFcγRIIB on splenic B cells. The R^2 values were calculated for from the line of best fit (R^2 = 0.5394; p 0.0101).

FIGURE S3

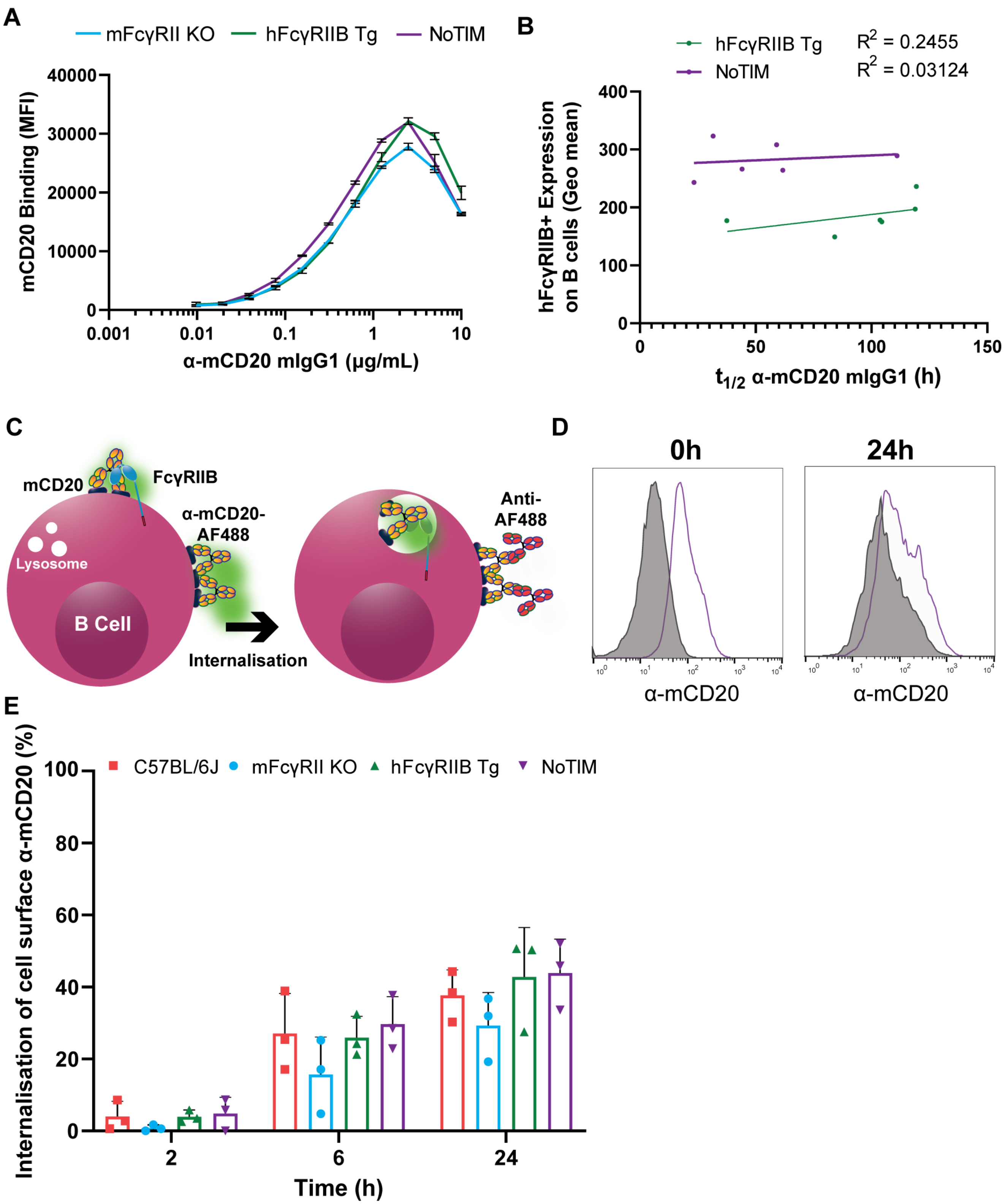

A
mFcγRII KO
hFcγRIIB Tg
NoTIM
mCD20 Binding (MFI)
40000
30000
20000
10000
0
0.001
0.01
0.1
1
10
α-mCD20 mIgG1 (μg/mL)
B
hFcγRIIB Tg
NoTIM
R2 = 0.2455
R2 = 0.03124
hFcγRIIB+ Expression on B cells (Geo mean)
400
300
200
100
0
0
50
100
150
t1/2 α-mCD20 mIgG1 (h)
C
mCD20
FcγRIIB
α-mCD20-AF488
Lysosome
B Cell
Internalisation
Anti-AF488
D
0h
24h
α-mCD20
α-mCD20
E
C57BL/6J
mFcγRII KO
hFcγRIIB Tg
NoTIM
Internalisation of cell surface α-mCD20 (%)
100
80
60
40
20
0
2
6
24
Time (h)

Figure S3, related to Figure 4. Impairment of B cell depletion in NoTIM mice is not due to insufficient serum levels or rapid internalization of mAb. A) Titration and saturation binding curve of anti-mCD20 (α-mCD20) mIgG1 binding to mouse B cells from mFcγRII$^{-/-}$, hFcγRIIB Tg and NoTIM mice; indicating a saturation level of ~2.5 µg/ml in each case. B) Relationship between hFcγRIIB Tg expression and $t_{1/2}$ in hFcγRIIB Tg and NoTIM mice. No clear correlation was observed, indicating that FcγRIIB expression does not influence α-mCD20 mAb serum levels. C, D and E) Splenic B cells were isolated from WT, mFcγRII$^{-/-}$, hFcγRIIB Tg and NoTIM mice and incubated in vitro with anti-mCD20-Alexa 488 mIgG1 for 2, 6 or 24 hours. Cells were then washed and then incubated in the presence or absence of anti-Alexa 488 quenching mAb as indicated in the schematic (C). D) indicates representative data after 0 and 24h. The fluorescence remaining after quenching indicates the proportion of internalized mAb, with % internalization calculated, and results plotted from 3 independent experiments in E) (conducted with triplicate samples). Columns represent means (+SD). Red: C57BL/6J WT; mFcγRII$^{-/-}$: blue; hFcγRIIB Tg: green; and NoTIM: purple. The data show that the presence of any FcγRIIB (mouse or human, WT or NoTIM) increases internalization but that this does not differ between the different FcγRIIB molecules.

FIGURE S4

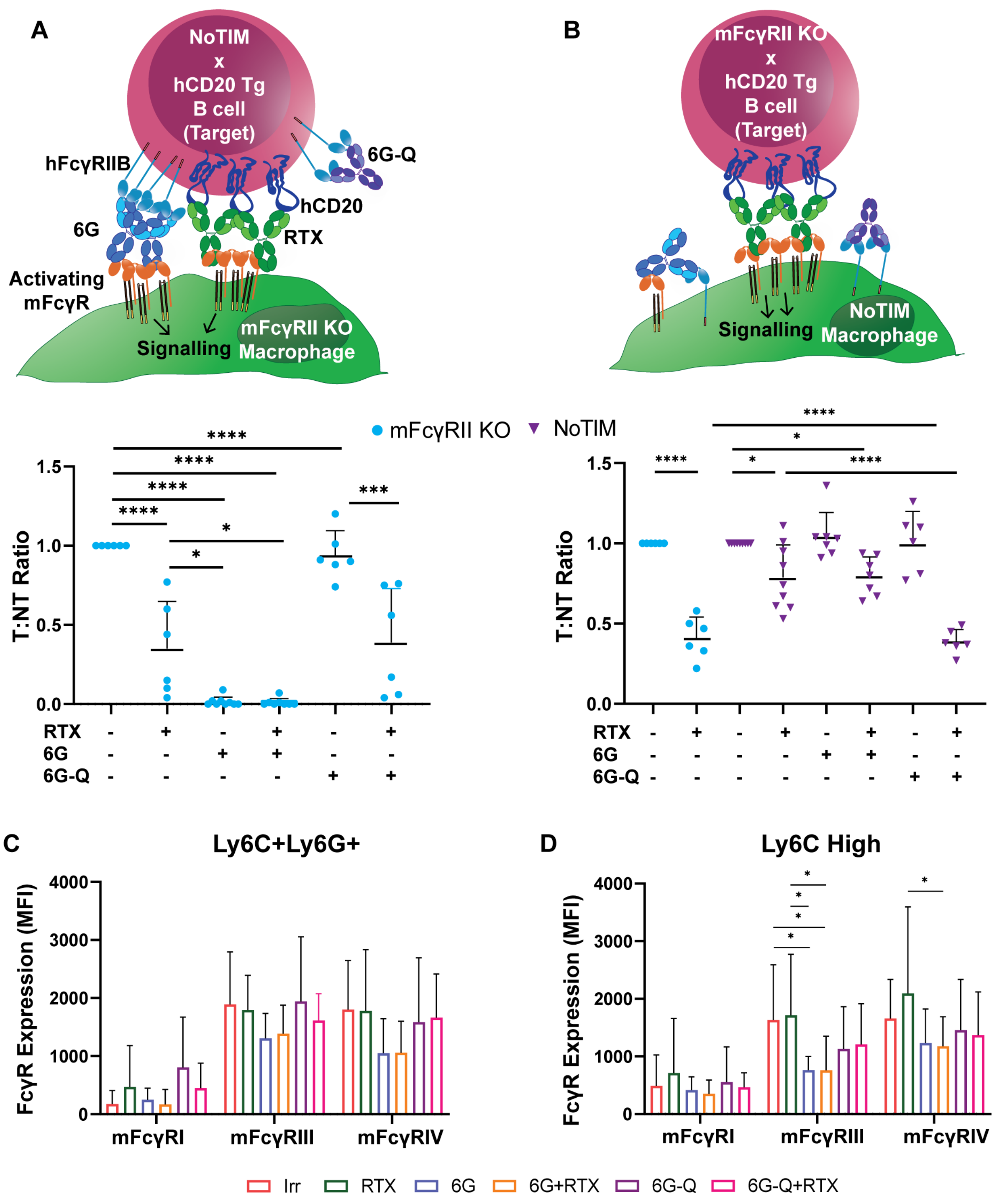
A
NoTIM
x
hCD20 Tg
B cell
(Target)
hFcγRIIB
6G-Q
hCD20
6G
RTX
Activating
mFcγR
mFcγRII KO
Macrophage
Signalling
B
mFcγRII KO
x
hCD20 Tg
B cell
(Target)
Signalling
NoTIM
Macrophage
mFcγRII KO
NoTIM
T:NT Ratio
RTX
6G
6G-Q
C
Ly6C+Ly6G+
FcγR Expression (MFI)
mFcγRI
mFcγRIII
mFcγRIV
D
Ly6C High
Irr
RTX
6G
6G+RTX
6G-Q
6G-Q+RTX

Figure S4, related to Figure 5. NoTIM hFcγRIIB impairs depletion through expression on effector cells not target cells. A) NoTIM hFcγRIIB^{+} hCD20^{+} mFcγRII$^{-/-}$ target (T) and NoTIM hFcγRIIB^{+} mFcγRII$^{-/-}$ hCD20^{-} non-target (NT) splenocytes were injected i.v. into mFcγRII$^{-/-}$ mice (as detailed in Fig. 5). Mice were treated as indicated and on day 2, spleens were taken and assessed for the expression of FcγRs at the surface of F4/80^{+} myeloid effector cells. The upper panel represents the schema, with data representing depletion of target cells within the bone marrow showing mean (+SD) from 2 independent experiments below. Statistical analyses were conducted using a one-way ANOVA with Tukey's multiple comparisons test. B) mFcγRII$^{-/-}$ x CD20 Tg target (T) and mFcγRII$^{-/-}$ non-target (NT) splenocytes were injected into NoTIM mice. Mice were treated as indicated and on day 3, spleens were taken and assessed for the expression of FcγRs at the surface of myeloid cells. Above represents the experimental schema with the depletion of target cells within the bone marrow below. Statistical analyses were conducted using a one-way ANOVA with Sidak's multiple comparisons test. C) represents summed expression of FcγRs on splenic Ly6C+Ly6G+ cells. D) is the summed data of expression on Ly6C High cells. Bar = mean (+SD) from 2-3 independent experiments. Statistical analyses were conducted using a one-way ANOVA with Tukey's multiple comparisons test. * $P \leq 0.05$, *** $P \leq 0.001$, **** $P \leq 0.0001$. In summary detection of mFcγRIII and IV on both Ly6G+Ly6C+ and Ly6C^{hi} cells was reduced by the presence of 6G (in the presence or absence of RTX) but not by 6G-Q (in the presence or absence of RTX) when the NoTIM is expressed only on the effector cells and not the targets.

FIGURE S5

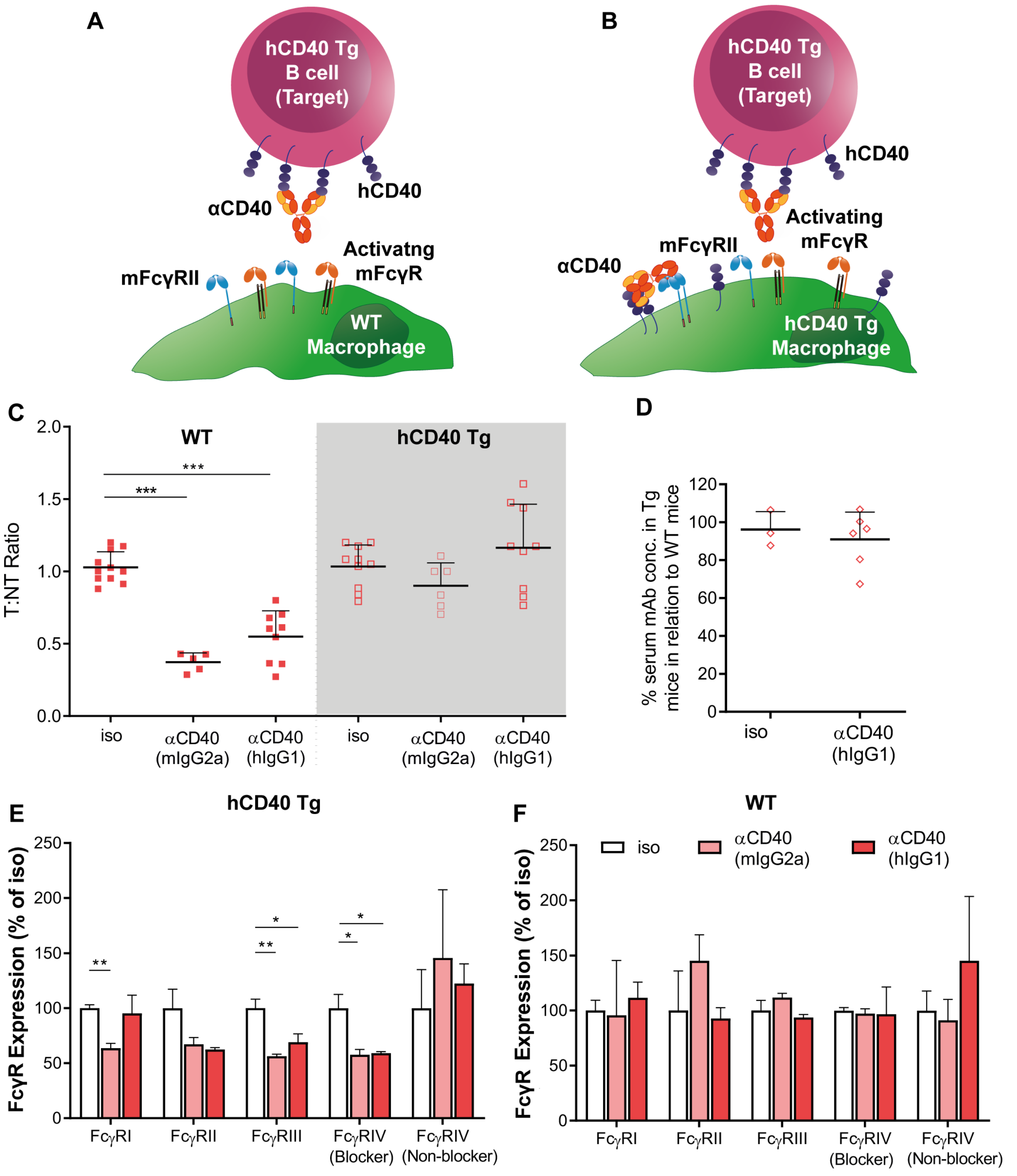
A
hCD40 Tg
B cell
(Target)
αCD40
hCD40
mFcγRII
Activatng
mFcγR
WT
Macrophage
B
hCD40 Tg
B cell
(Target)
hCD40
Activating
mFcγR
mFcγRII
αCD40
hCD40 Tg
Macrophage
C
WT
hCD40 Tg
T:NT Ratio
***
***
iso
αCD40 (mIgG2a)
αCD40 (hIgG1)
D
% serum mAb conc. in Tg mice in relation to WT mice
iso
αCD40 (hIgG1)
E
hCD40 Tg
FcγR Expression (% of iso)
**
*
**
*
*
FcγRI
FcγRII
FcγRIII
FcγRIV (Blocker)
FcγRIV (Non-blocker)
F
WT
iso
αCD40 (mIgG2a)
αCD40 (hIgG1)
FcγR Expression (% of iso)

Figure S5, related to Figure 5. Concurrent expression of hCD40 on target and effector cells limits target cell depletion. (A, B) hCD40 Tg (T) and WT (NT) splenocytes were injected into WT (as indicated in panel A) or hCD40 Tg recipient mice (as indicated in panel B) on day 0. Next day, mice received anti-human CD40 (αCD40) mAb, mIgG2a or hIgG1 and the following day the ratio of splenic $CD19^+$ cells were determined, as before (C; data combined from 2-4 independent experiments, each point represents a single mouse (n = 6-10 mice/group). Results in WT mice indicated by filled symbols; left unshaded panel or in hCD40 Tg mice by open symbols; right grey panel. D) Serum was collected 1 day after injection of αCD40 mAb (hIgG1) and quantified by ELISA (n = 3-6 mice/group). E-F) Expression of indicated activating and inhibitory FcγRs was assessed on splenic F4/80+ macrophages in (E) hCD40 Tg and (F) WT mice (n = 5 mice/group). Bars represent mean (+SD). * $P \leq 0.05$, ** $P \leq 0.01$.

FIGURE S6

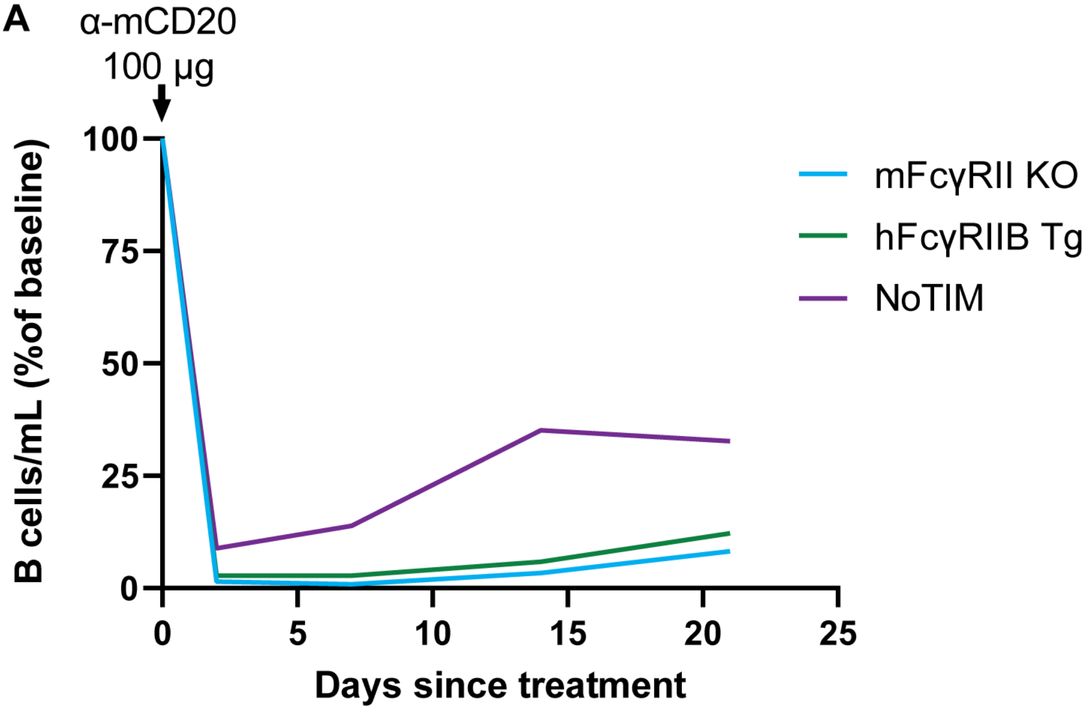

A
α-mCD20
100 µg
100
75
50
25
0
B cells/mL (%of baseline)
0
5
10
15
20
25
Days since treatment
mFcγRII KO
hFcγRIIB Tg
NoTIM

Figure S6, related to Figure 7. NoTIM hFcγRIIB impairs depletion of B cells in the Eu-TCL1 model. mFcγRII$^{-/-}$, hFcγRIIB Tg or NoTIM mice were injected with $5x10^6$ Eμ-TCL1 cells and monitored for tumor growth. When tumor equated to 10-20% of peripheral lymphocytes, mice were treated with 100 µg anti-mCD20 (α-mCD20) mIgG2a or isotype control. Flow cytometry was used to assess depletion of both normal B cells (CD19$^+$ B200^{hi} CD5$^-$); plotted is the kinetics of B cell depletion in each mouse model following treatment with mAb. Line = mean. The result of 3 independent experiments (n = 8-9 mice per group), The data shows that normal B cells are effectively deleted from the periphery in the presence of the Eμ-TCL1 cells at day 2 in all mice but that the B cells in the NoTIM mice recover more rapidly back to normal levels compared to mFcγRII$^{-/-}$ mice with hFcγRIIB Tg mice intermediate.
