# Supplementary material for: FcγRIIB controls antibody-mediated target cell depletion by ITIM-independent mechanisms
Source: Cell Rep. 2022 Jul 19;40(3):111099. doi: 10.1016/j.celrep.2022.111099 (PMC9638011; doi:10.1016/j.celrep.2022.111099)
Supplement: Document S2. Article plus supplemental information [file mmc2.pdf]

Cell Reports

Article

FcγRIIB controls antibody-mediated target cell depletion by ITIM-independent mechanisms

Graphical abstract

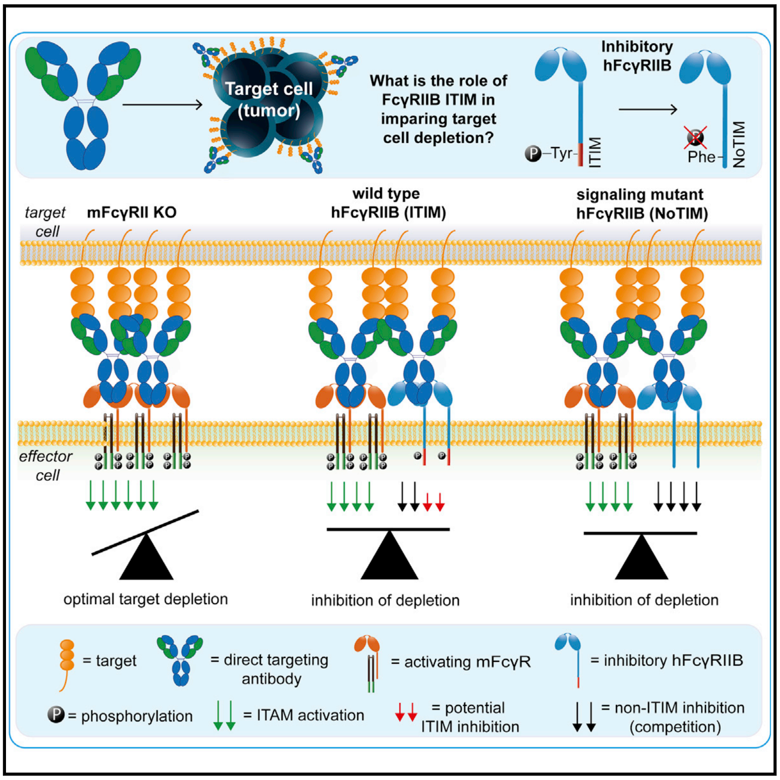

Highlights

- WT but not Fc-null FcγRIIB antibodies deplete FcγRIIB$^+$ targets
- ITIM signaling is dispensable for FcγRIIB-mediated inhibition of target cell depletion
- ITIM signaling is dispensable for FcγRIIB-mediated impairment of anti-tumor efficacy
- FcγRIIB inhibits mAb depletion by outcompeting activating FcγR on myeloid effectors

Authors

Alexander P. Simpson, Ali Roghanian, Robert J. Oldham, ..., Björn Frendeus, Stephen A. Beers, Mark S. Cragg

Correspondence

bjorn.frendeus@bioinvent.com (B.F.), sab@soton.ac.uk (S.A.B.), msc@soton.ac.uk (M.S.C.)

In brief

Simpson et al. demonstrate that the inhibitory FcγR, hFcγRIIB, mediates its effects through competition with other FcγRs, rather than ITIM signaling, to impair antibody-mediated target cell depletion and immunotherapy. This knowledge helps guide the development of next-generation anti-FcγRIIB reagents and optimal antibody immunotherapy strategies for the clinic.

Simpson et al., 2022, Cell Reports *40*, 111099
July 19, 2022 © 2022 The Author(s).
https://doi.org/10.1016/j.celrep.2022.111099

CellPress

Cell Reports

CellPress
OPEN ACCESS

Article

FcγRIIB controls antibody-mediated target cell depletion by ITIM-independent mechanisms

Alexander P. Simpson,[1,6] Ali Roghanian,[1,4,6] Robert J. Oldham,[1] H.T. Claude Chan,[1] Christine A. Penfold,[1] Hyung J. Kim,[1] Tatyana Inzhelevskaya,[1] C. Ian Mockridge,[1] Kerry L. Cox,[1] Yury D. Bogdanov,[1] Sonya James,[1] Alison L. Tutt,[1] Daniel Rycroft,[2] Peter Morley,[2] Lekh N. Dahal,[1,5] Ingrid Teige,[3] Björn Frendeus,[3,7,*] Stephen A. Beers,[1,7,*] and Mark S. Cragg[1,4,7,8,*]

[1]Antibody and Vaccine Group, Centre for Cancer Immunology, School of Cancer Sciences, University of Southampton Faculty of Medicine, Southampton SO16 6YD, UK

[2]Biopharm Discovery, GSK, Gunnels Wood Road, Stevenage SG1 2NY, UK

[3]BioInvent International AB, Sölvegatan 41, 22370 Lund, Sweden

[4]Institute for Life Sciences, University of Southampton, Southampton SO17 1BJ, UK

[5]Present address: Molecular and Clinical Pharmacology, Institute of Translational Medicine, Liverpool University, Liverpool, UK

[6]These authors contributed equally

[7]Senior author

[8]Lead contact

*Correspondence: bjorn.frendeus@bioinvent.com (B.F.), sab@soton.ac.uk (S.A.B.), msc@soton.ac.uk (M.S.C.)

https://doi.org/10.1016/j.celrep.2022.111099

SUMMARY

Many therapeutic antibodies deplete target cells and elicit immunotherapy by engaging activating Fc gamma receptors (FcγRs) on host effector cells. These antibodies are negatively regulated by the inhibitory FcγRIIB (CD32B). Dogma suggests inhibition is mediated through the FcγRIIB immunoreceptor tyrosine-based inhibition motif (ITIM), negatively regulating immunoreceptor tyrosine-based activation motif (ITAM)-mediated signaling from activating FcγR. To assess this, we generated experimental models expressing human (h) FcγRIIB on targets or effectors, lacking or retaining ITIM signaling capacity. We demonstrate that signaling through the hFcγRIIB ITIM is dispensable for impairing monoclonal antibody (mAb)-mediated depletion of normal and malignant murine target cells through three therapeutically relevant surface receptors (CD20, CD25, and OX40) affecting immunotherapy. We demonstrate that hFcγRIIB competition with activating FcγRs for antibody Fc, rather than ITIM signaling, is sufficient to impair activating FcγR engagement, inhibiting effector function and immunotherapy.

INTRODUCTION

Monoclonal antibodies (mAbs) such as rituximab, cetuximab, and trastuzumab are an important class of therapeutics, binding directly to tumor cells and evoking their destruction (Scott et al., 2012; Chan and Carter, 2010). Their mechanism of action is governed by interactions with Fc gamma receptors (FcγRs) (Glennie et al., 2007), with the response modulated by the specific receptor(s) engaged, the Fc valency of the immune complex (IC), the cell types involved, and architecture of the cellular microenvironment (DiLillo and Ravetch, 2015; Koenderman, 2019).

FcγRs can be activating or inhibitory (Bruhns, 2012). Activating FcγRs evoke immunoreceptor tyrosine-based activation motif (ITAM)-mediated signaling to elicit functions such as phagocytosis and cytokine release in response to antibody-coated cells or IC (Getahun and Cambier, 2015). Elegant studies highlight the significance of activating FcγRs in mediating therapeutic mAb activity, with loss of FcγR expression (Clynes et al., 2000) or signaling (de Haij et al., 2010) abrogating mAb-mediated cell depletion. In mice at least, the key FcγR-expressing effector cells derive from the mononuclear phagocyte system, mediating mAb-mediated target cell depletion for a range of targets, including CD20, EGFR, gp75, CD25, and OX40 (Beers et al., 2010; Grandjean et al., 2016; Gul et al., 2014; Roghanian et al., 2019; Setiady et al., 2010; Arce Vargas et al., 2017; Bulliard et al., 2014).

The counterpoint to the activating FcγR is the sole inhibitory FcγR, FcγRIIB (FcγRII in mice) (Roghanian et al., 2018). It contains an intracellular immunoreceptor tyrosine-based inhibition motif (ITIM), which, upon phosphorylation, acts to reverse the activating pathways initiated by ITAM-bearing receptors such as activating FcγR and the BCR (Getahun and Cambier, 2015). The phosphorylated ITIM provides a docking site for the phosphatases SHIP1 and SHP-1. These act to convert PIP_3 to PIP_2 and de-phosphorylate ITAM and ITAM-associated kinases such as Src and Syk (Huang et al., 2003) to attenuate activating FcγR signaling. FcγRIIB is also an important mediator of humoral immunity through regulation of ITAM signals downstream of the BCR, attenuating B cell activation, proliferation, survival, and differentiation as well as directly affecting affinity maturation and

This is an open access article under the CC BY license (http://creativecommons.org/licenses/by/4.0/).

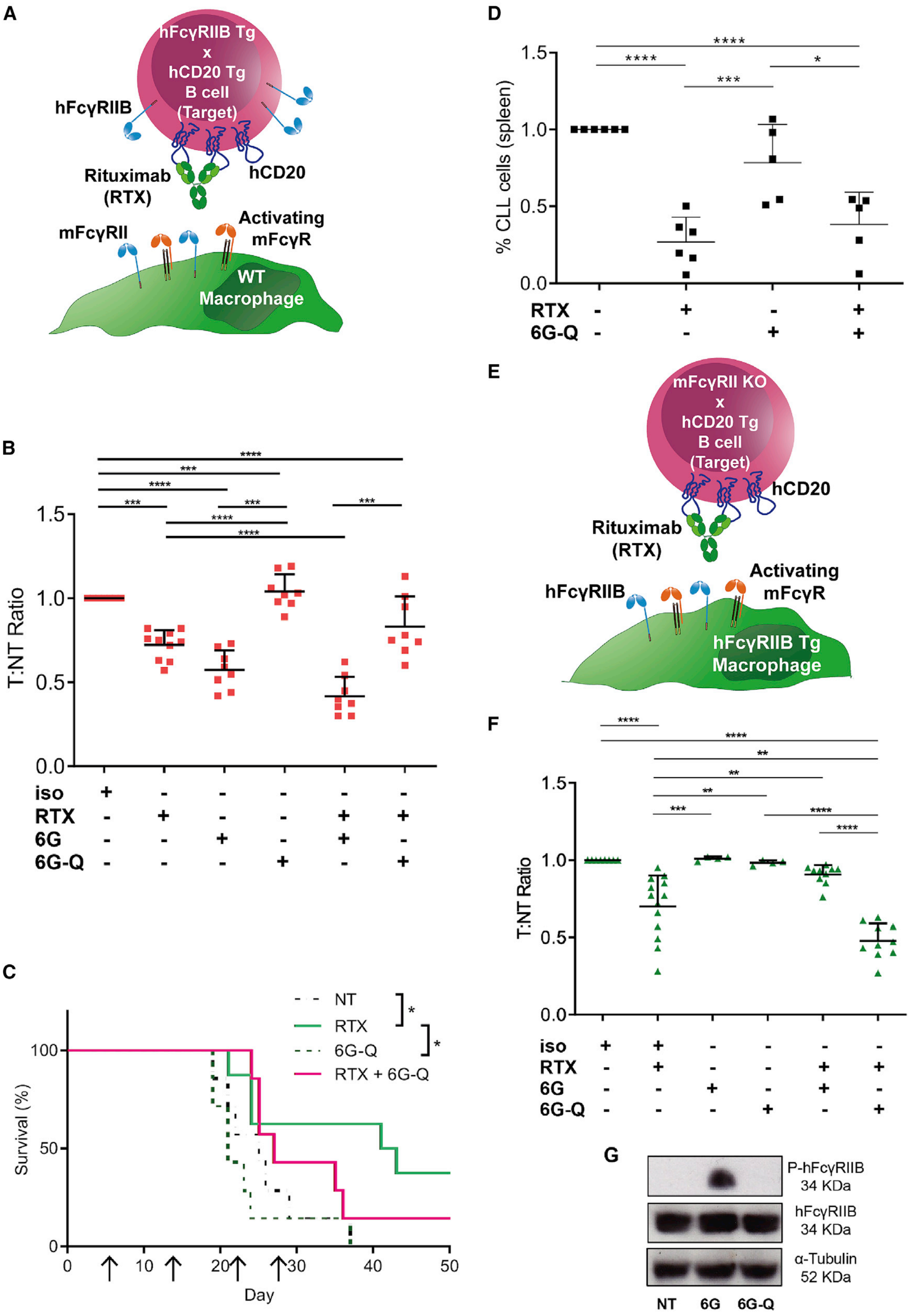

(legend on next page)

plasma cell survival (Ono et al., 1997; Xiang et al., 2007; Barrington et al., 2002).

FcγRIIB can negatively affect the therapeutic efficacy of mAb. For example, genetic deletion of mouse (m)FcγRII enhanced phagocytic function *in vitro* as well as mAb-mediated tumor control *in vivo* (Clynes et al., 1999, 2000). Pre-clinical and retrospective clinical studies also indicate that human (h)FcγRIIB has additional means of impairing direct targeting mAb. For example, B cell malignancies that demonstrate high expression of hFcγRIIB correlate with resistance to target cell depletion mediated by type-I anti-CD20 mAb, such as rituximab. Here, hFcγRIIB augments internalization of the CD20:mAb complex through *cis*-binding on the B cell surface (Lim et al., 2011), resulting in increased mAb consumption (Lim et al., 2011), attenuation of Fc-mediated effector functions (Beers et al., 2008; Tipton et al., 2015b) and reduced mAb persistence (Tipton et al., 2015b). Accordingly, high tumor cell hFcγRIIB is linked to poor response to rituximab in follicular lymphoma (Lee et al., 2015) and diffuse large B cell lymphoma (DLBCL) (Nowicka et al., 2021).

Together, these data support that hFcγRIIB represents an attractive target for overcoming resistance to direct targeting mAbs and improving therapeutic responses. We previously generated a panel of mAbs capable of targeting hFcγRIIB, showing they enhanced anti-CD20 mAb-mediated depletion of both normal and malignant B cells *in vivo*, as well as anti-CD52 mAb-mediated depletion of chronic lymphocytic leukemia (CLL) cells (Roghanian et al., 2015).

Although the importance of ITIM signaling has been established for the hFcγRIIB-mediated modulation of BCR signaling (Muta et al., 1994; Ono et al., 1997) and the inhibition of ITAM-related functions *in vitro* (Daeron et al., 1995), it has no role in the hFcγRIIB-mediated mAb internalization process, which is also independent of the hFcγRIIB intracellular tail (Vaughan et al., 2015). Similarly, the ability of hFcγRIIB to cluster and activate immunomodulatory mAbs is independent of hFcγRIIB signaling (Li and Ravetch, 2013; White et al., 2011). Crucially, the contribution of ITIM-mediated signaling to the inhibition of mAb-mediated cell depletion *in vivo* remains to be determined. To address this and better understand the most effective means of blocking FcγRIIB for enhancing direct targeting mAb efficacy, we generated a range of mouse models expressing human and/or mouse FcγRIIB on target or effector cells, lacking or retaining signaling capacity, alongside blocking reagents able to bind FcγRIIB alone or alongside other FcγR. We then deployed these tools to address these issues with respect to clinically relevant receptor targets on B cells and regulatory T cells (Tregs).

RESULTS

Impact of FcγRIIB mAbs on rituximab-mediated B cell depletion depends on Fc functionality and FcγRIIB expression profile

We previously developed a highly specific mAb directed to hFcγRIIB (6G11, 6G, BI-1206), that does not bind to mFcγRII, and showed it augmented rituximab-mediated depletion of normal and malignant B cells *in vivo* (Roghanian et al., 2015). We postulated activity was based on its ability to engage three separate mechanisms: (1) direct Fc:FcγR-mediated cell depletion (with hFcγRIIB operating as a direct targeting antigen), (2) prevention of rituximab internalization, and (3) blocking hFcγRIIB-mediated inhibition of myeloid effector cells (Roghanian et al., 2016). To delineate the relative importance of these and which FcγRIIB mAb format was optimal, we developed a panel of hFcγRIIB transgenic (Tg) mice and hFcγRIIB mAbs displaying wild-type (WT) or defective (N297Q mutation (herein referred to as Fc-null) Fc domains. We then assessed their ability to deplete $hCD20^{+}$ target B cells both alone and in combination with rituximab in models where hFcγRIIB was present on target cells, effector cells, or both. Using this approach, we were able to measure depletion of the adoptively transferred B cells or tumor cells in the presence or absence of different FcγRIIB molecules and investigate depletion with a clinically relevant anti-hCD20 mAb.

Initially, we tested systems where hFcγRIIB was expressed only on the target cell, adoptively transferring $hCD20^{+/-}$ × $hFc\gamma RIIB^{+/-}$ × $mFc\gamma RII^{-/-}$ murine splenocytes into WT C57BL/6J mice (Figure 1A). WT hFcγRIIB mAb (6G) depleted target cells when administered alone and potentiated the activity of rituximab (RTX) (Figure 1B). Fc-null hFcγRIIB mAb (6G-Q) did not deplete target B cells (due to the absence of a functional Fc) and did not improve depletion in combination with rituximab (Figure 1B). Next, we used a human Raji lymphoma xenograft model and showed that 6G-Q mAb inhibited the therapeutic activity of rituximab, abolishing tumor control (Figure 1C). These observations with 6G-Q in both systems were in contrast to our previous

Figure 1. Efficacy of CD20 mAbs in combination with FcγRIIB blockade depends on FcγRIIB mAb format

(A) Schematic of models used in (B)–(D) where hFcγRIIB is expressed only on target cells.

(B) $hFc\gamma RIIB^{+/-}$ × $mFc\gamma RII^{-/-}$ target (T) and $mFc\gamma RII^{-/-}$ non-target (NT) cells were adoptively transferred into WT recipient mice. Mice received mAb as indicated and splenic cells analyzed to determine the ratio of T:NT cells. Data from ≥3 independent experiments.

(C) Raji cells were injected into severe combined immune deficient (SCID) mice (n = 7 mice/group) and treated with indicated mAb weekly from day 7, up to four times. Survival analyzed using Kaplan-Meier and Log rank test.

(D) Anti-tumor activity of RTX, 6G-Q, or both in mice xenografted with human CLL cells (n = 2 patients; five or six mice). Mice were treated with either 1–10 mg/kg of RTX, 6G-Q, or both and percentage of CLL cells remaining in the spleen was enumerated.

(E) Schematic of models used in (F) where hFcγRIIB is expressed only on the effector cells.

(F) $hCD20^{+/-}$ × $mFc\gamma RII^{-/-}$ (T) and $mFc\gamma RII^{-/-}$ (NT) splenocytes were injected into $hFc\gamma RIIB^{+/-}$ × $mFc\gamma RII^{-/-}$ recipient mice. Mice received 6G or 6G-Q mAb (2 × 20 mg/kg) followed by RTX (0.2–2 mg/kg) and splenic cells were analyzed to determine the ratio of T:NT cells. Data combined from ≥2 independent experiments.

(G) Ability of 6G or 6G-Q to elicit hFcγRIIB ITIM phosphorylation (P-FcγRIIB) on BMDMs. α-Tubulin and hFcγRIIB loading controls.

(B, D, F) Each dot depicts a result from a single mouse, lines represent mean (+SD). Statistical analyses: one-way ANOVA with Tukey's multiple comparisons. $^{*}p \leq 0.05$, $^{**}p \leq 0.01$, $^{***}p \leq 0.001$, $^{****}p \leq 0.0001$.

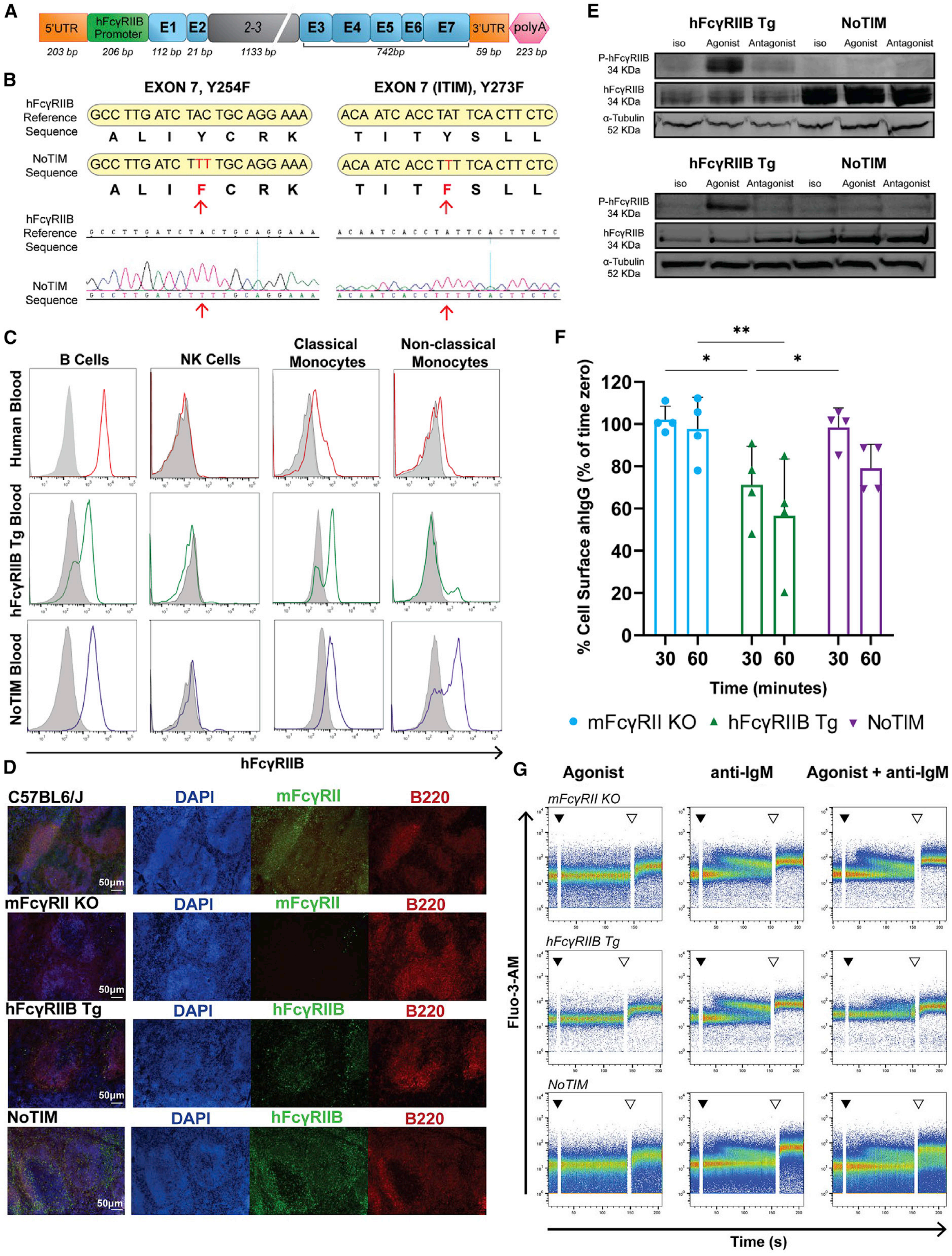

(legend on next page)

findings with 6G in combination with rituximab where we demonstrated a clear beneficial effect (Roghanian et al., 2015). Finally, we tested the effects of 6G-Q + rituximab in a second xenograft model, primary human CLL cells, showing it did not improve the depletion compared with rituximab alone (Figure 1D).

Next, we assessed the impact of anti-hFcγRIIB mAb when hFcγRIIB was lacking from the target cells and expressed only on the effector cells. Target hCD20$^{+/-}$ × mFcγRII$^{-/-}$ murine splenocytes (lacking hFcγRIIB) were adoptively transferred into hFcγRIIB$^{+/-}$ × mFcγRII$^{-/-}$ recipients and treated as before (Figure 1E). 6G and 6G-Q mAb alone had no effect on target B cells, whereas rituximab monotherapy resulted in a ~40% reduction (Figure 1F). In direct contrast to that observed in Figures 1B–1D, 6G-Q potentiated the depletion of B cells when combined with rituximab, whereas 6G impaired activity. Together, these observations demonstrate that the Fc functionality of hFcγRIIB mAb has differential and opposing effects on augmenting B cell depletion when directed to target or effector cells. Given the opposing effects of the two hFcγRIIB mAb formats, we next addressed whether they had the potential to stimulate ITIM signaling in relevant immune effector cells. Bone marrow-derived macrophages (BMDMs) were stimulated with either 6G or 6G-Q and phosphorylation of the FcγRIIB ITIM domain assessed. Treatment with 6G, but not 6G-Q, resulted in the phosphorylation of the hFcγRIIB-ITIM (Figure 1G). These data could explain why WT hFcγRIIB mAbs but not Fc-null mAbs impair the depleting capacity of rituximab (by delivering hFcγRIIB-mediated inhibitory signaling into myeloid cells). However, this assumes that ITIM-mediated signaling is central to these inhibitory effects and so we explored this directly by generating hFcγRIIB Tg mice carrying a defective ITIM.

Generation and characterization of hFcγRIIB NoTIM mice, lacking a functional ITIM

ITIM-defective mice were generated based upon our previous hFcγRIIB Tg construct (Roghanian et al., 2015) with mutation of Y273 within the ITIM to phenylalanine (Y273F), which impairs interaction with SHIP-1 (Stopforth et al., 2018). To further reduce the potential for signaling we also mutated Y254F in exon 7, proximal to the ITIM (Figure 2A), confirmed by Sanger sequencing (Figures 2B, S1A, and S1B). We termed this model NoTIM in reference to the non-signaling activating FcγR NOTAM mouse produced previously (de Haij et al., 2010) and crossed it with mFcγRII$^{-/-}$ mice to remove the endogenous mouse inhibitory FcγR. Surface expression of the NoTIM hFcγRIIB was confirmed by flow cytometry (FCM) and immunofluorescence microscopy on relevant cell populations; i.e., B cells and monocytes but not natural killer (NK) cells (Figures 2C and 2D). Western blot using an agonistic hFcγRIIB mAb (6G08) (Roghanian et al., 2015) demonstrated that hFcγRIIB ITIM phosphorylation was lost in NoTIM BMDMs and B cells (Figure 2E). Further analysis showed phosphorylated SHIP-1 in hFcγRIIB Tg B cells but not B cells from NoTIM mice (Figure S1E). NoTIM B cells were also partially impaired in their ability to internalize soluble ICs compared with B cells expressing WT hFcγRIIB (Figure 2F), in agreement with earlier studies showing mFcγRII regulates IC internalization and loss of its ITIM tyrosines reduced the speed of internalization (Miettinen et al., 1992). The same agonistic hFcγRIIB mAb was also able to reduce anti-immunoglobulin (Ig)M-mediated BCR calcium flux in hFcγRIIB Tg but not NoTIM B cells (Figure 2G). Having established a lack of inhibitory FcγR signaling in these mice, next, we assessed their full FcγR expression pattern and found hFcγRIIB expression to be comparable with that in our hFcγRIIB Tg mouse (and in humans; Figures 2C, S1C, and S1D), without significant change to activating mFcγRs (Figures S1F and S1H). Together, these data demonstrate that expression of a non-signaling hFcγRIIB was achieved in NoTIM mice in a physiologically relevant manner akin to that in mice expressing a functional hFcγRIIB, facilitating subsequent comparisons.

hFcγRIIB-mediated inhibition of anti-mCD20 mAb is not dependent on inhibitory signaling *in vivo*

To determine if ITIM signaling was important for impairing anti-mCD20-mAb-mediated B cell depletion, mice were treated with increasing, stepwise doses (2, 10, 50 μg) of an anti-mCD20 mAb (clone: 18B12) (Ahuja et al., 2007). Both mIgG1 and mIgG2a isotypes were utilized due to their differing activating to inhibitory (A:I) FcγR binding profiles and ratios (Nimmerjahn and Ravetch, 2005), with depletion of circulating B cells then assessed (Figures 3A and 3B). When using mIgG1, with its modest A:I ratio (engaging only mFcγRII and mFcγRIII), mFcγRII$^{-/-}$ mice (expressing only activating mFcγRs) were most susceptible to mAb-mediated B cell depletion (Clynes et al., 2000) (Figure 3C). Notably, NoTIM mice, lacking intrinsic inhibitory signaling, were significantly

Figure 2. Generation and characterization of the NoTIM mouse

(A) Schematic representation of the transgene construct used to generate NoTIM mice; hFcγRIIB promoter, exons (E) 1–2, introns 2–3, and exons 3–7.

(B) Schematic and DNA sequencing profiles of the ITIM region in the NoTIM mouse showing substitution of Y for F. Red arrows indicate point mutations confirmed by Sanger sequencing.

(C) hFcγRIIB expression assessed on circulating B cells, NK cells, and monocytes from blood using FCM. Representative histograms from three independent experiments.

(D) Frozen sections from mouse spleens were evaluated by immunofluorescence for expression of hFcγRIIB and mFcγRII.

(E) BMDMs (upper) or splenic B cells (lower) were isolated from NoTIM or hFcγRIIB Tg mice, stimulated with hFcγRIIB-specific mAbs and probed for hFcγRIIB ITIM phosphorylation (P-FcγRIIB) and total hFcγRIIB. α-tubulin loading control.

(F) Splenic B cells isolated from mFcγRII$^{-/-}$, hFcγRIIB Tg, or NoTIM mice were cultured with heat aggregated IgG (ahIgG). The proportion of total ahIgG remaining on the cell surface after 30 and 60 min was assessed by FCM. n = 3. Horizontal bars represent the mean + SD.

(G) Splenic B cells were isolated from mFcγRII$^{-/-}$, hFcγRIIB Tg, or NoTIM mice, labeled with Fluo-3-AM and pre-incubated with an hFcγRIIB agonist or isotype control. Samples were then analyzed by FCM before and after the addition of F(ab′)$_2$ anti-IgM. Black arrow indicates the addition of F(ab′)$_2$ anti-IgM. White arrow shows addition of 0.6 μM ionomycin (positive control). Statistical analyses: one-way ANOVA with Tukey's multiple comparisons. *p ≤ 0.05, **p ≤ 0.01, ***p ≤ 0.001, ****p ≤ 0.0001. See also Figure S1.

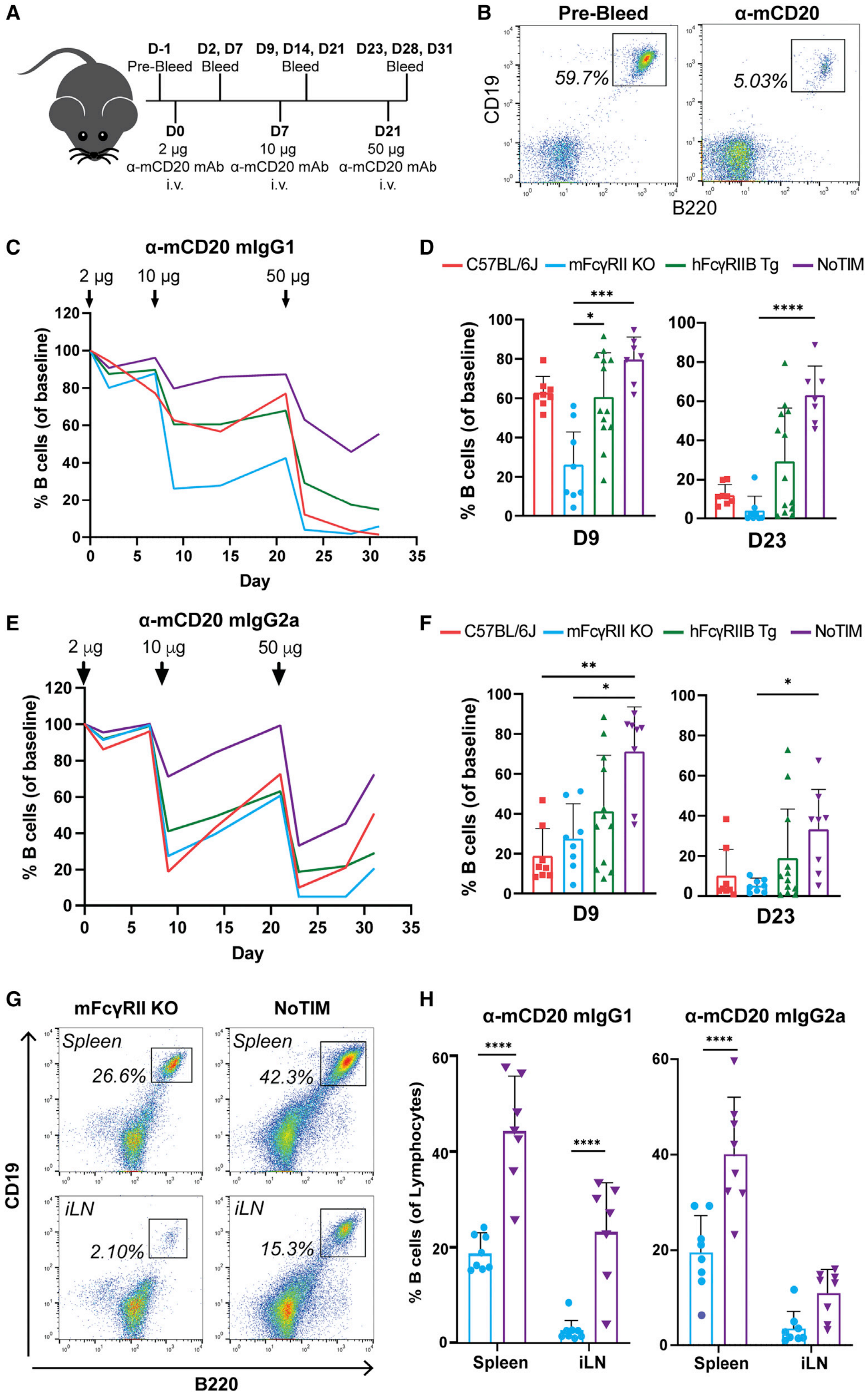

(legend on next page)

more resistant to anti-mCD20-mediated B cell depletion than mFcγRII$^{-/-}$ mice, with hFcγRIIB Tg and WT C57BL/6J mice displaying an intermediate response after both 10-μg and 50-μg doses (assessed on day 9 and day 23, respectively; Figure 3D). This difference was most evident after the final 50-μg dose on day 23. The signaling-competent hFcγRIIB Tg mice formed two clusters: responders displaying B cell depletion similar to mFcγRII$^{-/-}$ mice and non-responders showing depletion more similar to NoTIM mice (Figure 3D). As previously observed (Roghanian et al., 2015), our hFcγRIIB Tg mice display a mosaic expression pattern (Figure S1G), with penetrance varying between mice. To assess if Tg expression affected depletion, the percentage hFcγRIIB positivity of each mouse was quantified using FCM and then correlated with peripheral B cell depletion. There was a strong correlation between Tg expression and percentage of remaining peripheral B cells in these mice, indicating a direct relationship between hFcγRIIB expression and inhibition of anti-mCD20-mediated B cell depletion (Figure S2).

mIgG2a additionally engages mFcγRI and mFcγRIV, displaying a higher FcγR A:I ratio than mIgG1. Accordingly, B cells were depleted more effectively at lower doses with anti-mCD20 mIgG2a in WT mice than the mIgG1 mAb, with WT and mFcγRII$^{-/-}$ mice displaying similar levels of depletion (Figures 3E and 3F). Nevertheless, clear inhibition of B cell depletion was seen in NoTIM mice, with hFcγRIIB Tg mice displaying intermediate inhibition. The impacts on depletion for anti-mCD20 mIgG1 and mIgG2a were also reflected in the number of B cells observed in the spleen and lymph nodes (Figures 3G and 3H), with depletion significantly reduced in NoTIM mice. These data demonstrate that cell surface expression of hFcγRIIB is tightly linked to inhibition of anti-mCD20 mAb-mediated B cell depletion and that ITIM-mediated signaling is not required for impairing the depletion capabilities of mIgG1 or mIgG2a isotypes.

hFcγRIIB does not adversely affect anti-mCD20 mAb serum exposure

FcγRIIB can regulate antibody half-life through the removal of small ICs, predominantly via the sinusoidal endothelial cells of the liver (Ganesan et al., 2012). To ascertain if the lack of depletion in the various mouse strains was due to rapid loss of anti-mCD20 mAb from circulation, serum exposure was assessed. mFcγRII$^{-/-}$, hFcγRIIB Tg, and NoTIM mice were intravenously injected with 50 μg of mAb and bled to ascertain serum concentrations for comparison with WT C57BL/6J mice (Figure 4A). B cell depletion was concurrently assessed. Serum mAb levels remained similar across strains (Figures 4B and 4C) and did not correlate with depletion at 6 or 48 h (Figures 4C and 4D). The observed mAb half-life ($t_{1/2}$), area under the curve ($AUC_{0\text{-}t}$), volume of distribution (V_z), and clearance (CL) indicated serum availability was similar across all groups (Figure 4E). Although serum levels remained highest in mFcγRII$^{-/-}$ mice, the nadir of all groups remained above that required for saturation of mCD20 on B cells at 48 h post injection (Figure S3A). Together, these data indicate that anti-mCD20 serum exposure does not explain the difference in depletion efficacy between the four mouse models. Further supporting this notion, mAb exposure was independent of Tg expression in hFcγRIIB Tg mice (Figure S3B).

hFcγRIIB regulates anti-mCD20 mAb internalization independently of depletion

Antibodies directed to human CD20 can undergo internalization, which is accelerated by co-engagement of hFcγRIIB (Lim et al., 2011), but independent of intracellular signaling (Vaughan et al., 2015). We therefore considered whether rapid internalization of mAb-mCD20 complexes from the B cells might explain why NoTIM mice were highly resistant to B cell depletion. An *in vitro* internalization assay using B cells from each mouse model (Figures S3C and S3D) showed internalization was modest overall, being near absent at 2 h and ~40% after 24 h. Although broadly similar levels of internalization were seen, the presence of the mouse or human inhibitory receptor (C57BL/6J, hFcγRIIB, or NoTIM) accelerated the internalization of anti-mCD20 mIgG1 to a similar degree, compared with the mFcγRII$^{-/-}$ B cells (Figure S3E). These results are in keeping with previous observations, showing that hFcγRIIB increases internalization of anti-hCD20 mAb from the B cell surface (Beers et al., 2010; Lim et al., 2011; Vaughan et al., 2014). Consequently, this may explain the small differences in serum exposure between mFcγRII$^{-/-}$ and other mouse models but is unlikely to explain the profound differences in B cell depletion observed.

NoTIM effector cells suppress anti-hCD20 mAb-mediated target cell depletion *in vivo*

As described earlier, hFcγRIIB is expressed on both B cell targets and myeloid effector cells. To establish the cell type on which FcγRIIB exerts its inhibitory effects, we devised experiments to separately examine its impact on target or effector cells (akin to those in Figure 1). To determine the effects of hFcγRIIB on targets, hCD20$^{+/-}$ × NoTIM splenocytes (targets [T]) were adoptively transferred alongside mFcγRII$^{-/-}$ splenocytes (non-targets [NT]) into mFcγRII$^{-/-}$ recipients (Figure 5A). Mice were

Figure 3. Anti-mouse CD20 mAb-mediated depletion of B cells is impaired in NoTIM mice

(A) WT, mFcγRII$^{-/-}$, hFcγRIIB Tg, or NoTIM mice were treated with escalating doses of 18B12 mIgG1 or mIgG2a and bled to ascertain B cell depletion kinetics; D, day.

(B) Gating strategy for B cells; CD19^{+}B220^{+}.

(C and D) Composite data from (A) using 18B12 mIgG1; line graph indicates average percentage depletion of B cells in each mouse strain (C) with individual time points shown in (D); each point represents a different mouse (n = 7–13 mice/group). Result of two independent experiments. Each column represents the mean (+SD). Statistical analyses: Kruskal-Wallis test with Dunn's multiple comparisons test.

(E and F) As (C) and (D) but with 18B12 mIgG2a.

(G and H) On day 31 (D31) spleens and inguinal lymph nodes (iLNs) harvested from mFcγRII$^{-/-}$ and NoTIM mice and analyzed by FCM to assess B cell depletion.

(G) Representative flow plots and (H) bar graphs indicating the mean percentage of remaining B cells (+SD). Each point represents one mouse (n = 7–13 mice/group). Result of two independent experiments. Statistical analyses: one-way ANOVA with Sidak's multiple comparisons. *p ≤ 0.05, **p ≤ 0.01, ***p ≤ 0.001, ****p ≤ 0.0001.

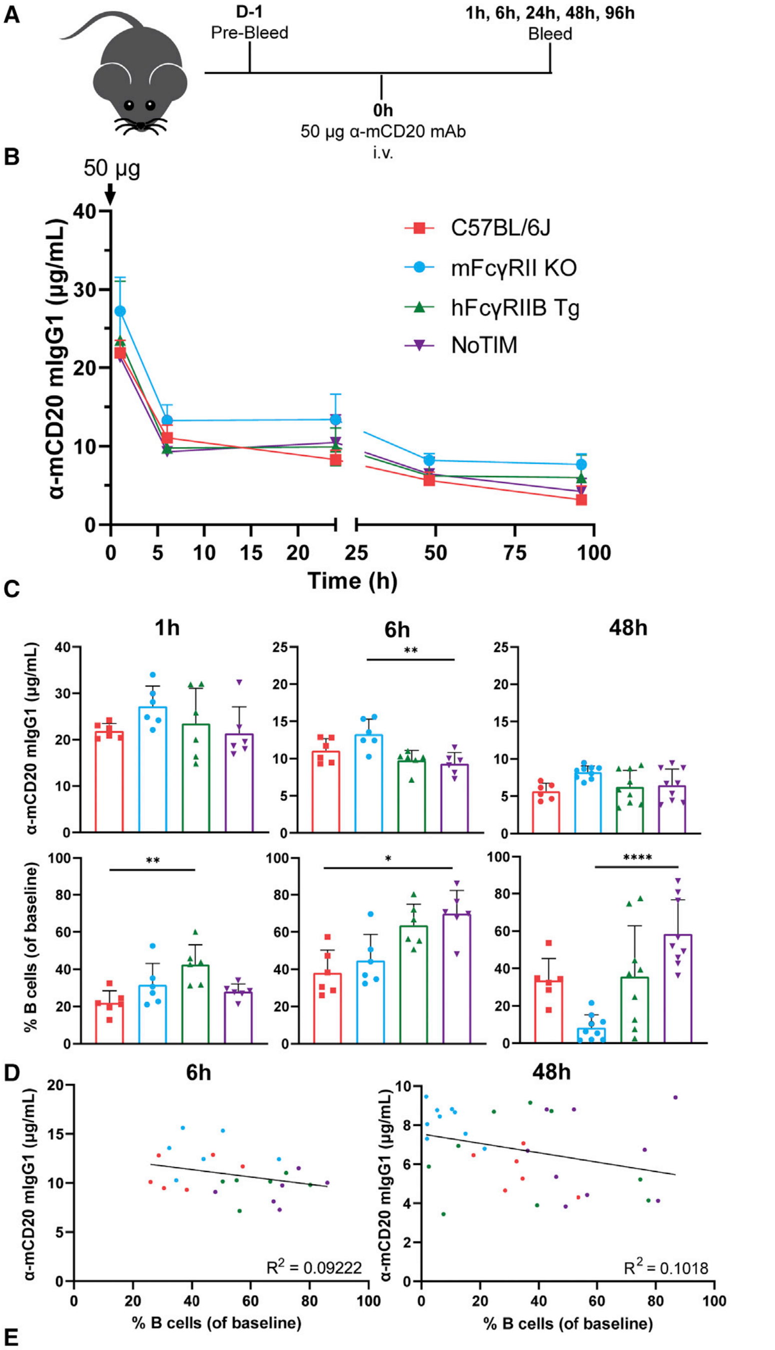

E

Parameter	Unit	C57Bl/6J mean ±SD (Range)	mFcγRII KO mean ±SD (Range)	hFcγRIIB Tg mean ±SD (Range)	NoTIM mean ±SD (Range)
$t_{1/2}$	h	39.52 ±12.64 (29.66 - 61.10)	63.06 ±10.96 (50.95 - 77.71)	64.08 ±22.05 (37.54 - 71.53)	47.99 ±16.57 (28.80 - 71.53)
$AUC_{0\text{-}t}$	µg/ml*h	662.13 ±68.72 (584.36 - 767.58)	991.72 ±88.04 (893.28 - 1123.39)	802.08 ±220.14 (491.22 - 1065.65)	723.20 ±167.73 (541.32 - 986.58)
V_z	(µg)/(µg/ml)	3.27 ±0.37 (2.80 - 3.47)	2.75 ±0.21 (2.60 - 2.99)	3.50 ±0.77 (2.48-4.42)	3.46 ±0.65 (3.15 - 4.21)
CL	(µg)/(µg/ml)/h	0.0607 ±0.0136 (0.0382 - 0.0751)	0.0309 ±0.0052 (0.0232 - 0.0366)	0.0429 ±0.0217 (0.0203 - 0.0816)	0.0541 ±0.0171 (0.0305 - 0.0766)

Figure 4. Impaired depletion in the NoTIM mouse is not related to differential antibody persistence

(A) WT, mFcγRII$^{-/-}$, hFcγRIIB Tg, or NoTIM mice were injected with 50 µg of 18B12 mIgG1 intravenously (i.v.) and bled over 96 h to ascertain the level of available mAb in the serum and percentage of B cells in the blood as indicated. Result of one to three independent experiments (n = 6–9 mice/group).

(B) Available serum 18B12 was measured and represented as the mean (+SD).

(C) Available serum 18B12 (top) and B cell depletion (lower) concurrently determined at 1, 6, and 48 h. Columns represent means (+SD); dots represent results from individual mice.

(D) Correlation between 18B12 serum levels at 6 h or 48 h and B cell depletion for the four different strains. See also Figure S3.

(E) Data in (B) and (C) were used to determine $t_{1/2}$, $AUC_{0\text{-}t}$, V_z, and CL parameters. (C) Statistical analyses: one-way ANOVA with Tukey's multiple comparison (serum availability) and Kruskal-Wallis test with Dunn's multiple comparisons (percentage of B cells). *$p \leq 0.05$, **$p \leq 0.01$, ****$p \leq 0.0001$.

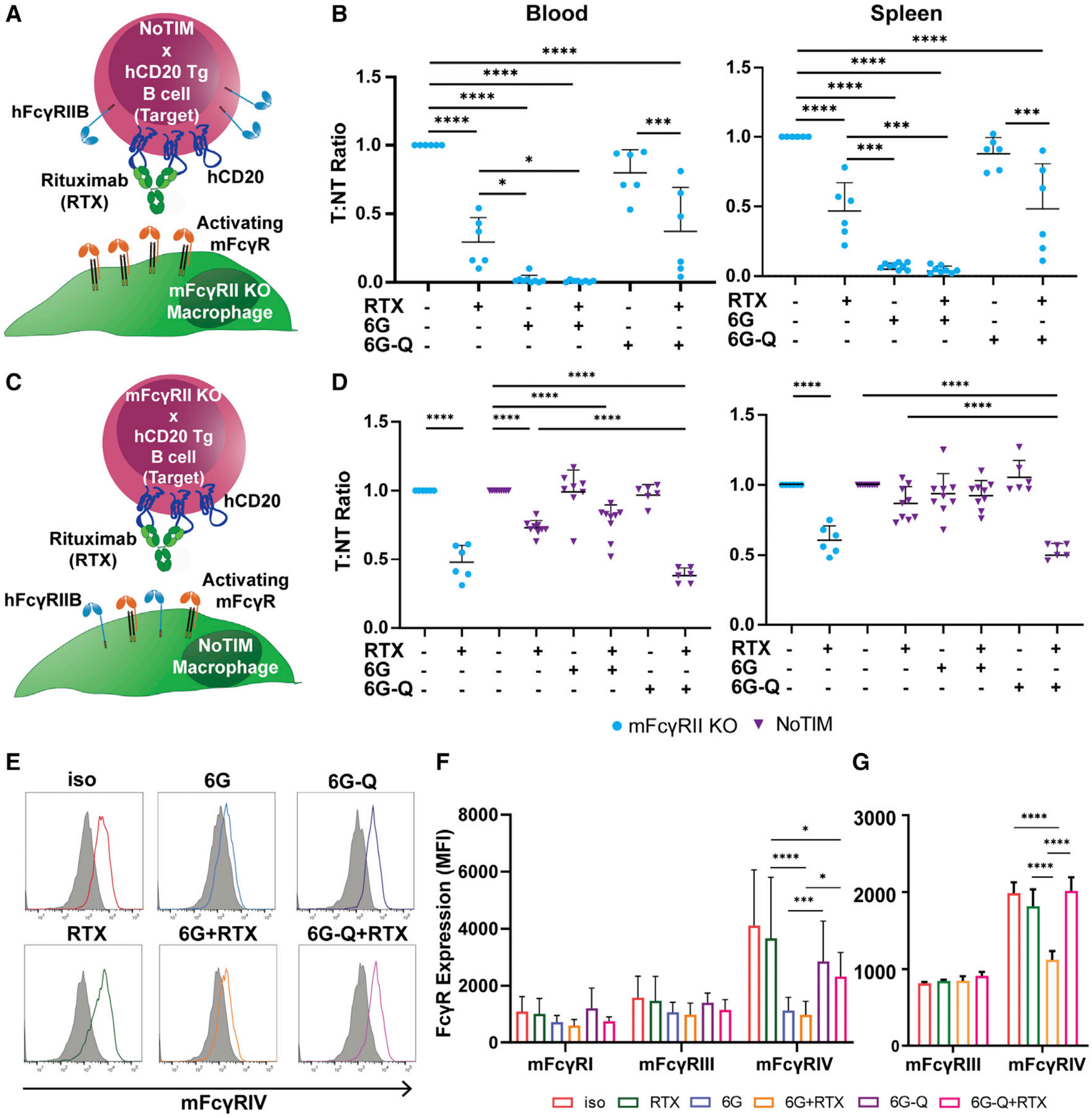

Figure 5. NoTIM hFcγRIIB impairs depletion through expression on effector cells

(A and B) NoTIM hFcγRIIB$^+$ hCD20$^+$ mFcγRII$^{-/-}$ (T) and NoTIM hFcγRIIB$^+$ mFcγRII$^{-/-}$ (NT) splenocytes were injected i.v. into mFcγRII$^{-/-}$ mice (schematic shown in A). One day later, mice received 2 mg/kg hFcγRIIB blocking antibody (6G, 6G-Q, or isotype) intraperitoneally (i.p.) alongside 2 mg/kg rituximab (RTX) (or isotype) i.v. On day 2, mice were analyzed for T:NT ratio in the blood, spleen, and bone marrow by FCM. Lines represent mean (+SD). Statistical analyses: one-way ANOVA with Tukey's multiple comparisons. Result of two or three independent experiments.

(C and D) mFcγRII$^{-/-}$ × CD20$^{-/+}$ (T) and mFcγRII$^{-/-}$ (NT) splenocytes were injected into mFcγRII$^{-/-}$ or NoTIM mice. On day 1, mice received 20 mg/kg hFcγRIIB blocking antibody (6G, 6G-Q, or isotype) i.p. and a further injection on day 2, alongside 2 mg/kg RTX (or isotype) i.v. On day 3, mice were analyzed for T:NT ratio by FCM as in B). The result of two independent experiments. Bars represent mean (+SD). Blue, mFcγRII$^{-/-}$ recipient mice; purple, NoTIM recipient mice. Statistical analyses: one-way ANOVA with Sidak's multiple comparisons.

(E and F) FCM was used to analyze the availability of activating mFcγRs on immune effector cells.

(E) Plots show the detection of mFcγRIV on splenic macrophages.

(F) The mean fluorescence intensity (MFI) of each mFcγR on splenic macrophages. Bars represent mean (+SD) from two or three independent experiments. Statistical analyses: one-way ANOVA with Tukey's multiple comparisons.

(G) mFcγR expression on splenic macrophages as in (F), from an equivalent adoptive transfer into WT hFcγRIIB Tg mice. Bars represent mean (+SD). See also Figure S4. *$p \leq 0.05$, **$p \leq 0.01$, ***$p \leq 0.001$, ****$p \leq 0.0001$

dosed with mAb to block FcγRIIB, with either WT (6G) or null (6G-Q) Fc domains, and then treated with rituximab before measuring the (T:NT) ratio. To assay for potential impacts in different locations, the blood, spleen, and bone marrow were examined. Rituximab resulted in robust depletion of target cells from the blood and spleen, with similar effects in the bone

marrow (Figures 5B and S4A, respectively). 6G also resulted in potent depletion of target B cells expressing the NoTIM hFcγRIIB, both alone and in combination with rituximab. In contrast, 6G-Q monotherapy had no impact on B cell depletion and did not improve target depletion in combination with rituximab. These data indicate that 6G enhances target cell depletion through opsonization of the hFcγRIIB$^+$ target cell. In contrast, 6G-Q, which cannot interact with activating mFcγRs, could not mediate or promote cell depletion of FcγRIIB$^+$ target cells (Figure S4A), as observed in WT hFcγRIIB mice (Figure 1D).

Next, we assessed the impact of the NoTIM hFcγRIIB on effector cells. Accordingly, splenocytes were isolated from hCD20$^{+/-}$ × mFcγRII$^{-/-}$ mice (T) and adoptively transferred alongside mFcγRII$^{-/-}$ splenocytes (NT) into mFcγRII$^{-/-}$ or NoTIM mice (Figure 5C). Mice were treated as before (Figures 1B–1D and 5B) and the T:NT ratio determined. As expected, rituximab monotherapy was effective in mFcγRII$^{-/-}$ mice (Figure 5D; blue circles). In contrast, rituximab monotherapy in NoTIM mice was less efficient, depleting fewer target B cells (purple triangles), indicating that inhibition of target cell depletion arises from expression of the NoTIM receptor on effector cells (Figures 5D and S4B). The addition of 6G to rituximab did not improve target cell depletion in NoTIM mice. However, the addition of 6G-Q significantly enhanced rituximab depletion compared with rituximab monotherapy to levels equivalent to those in mFcγRII$^{-/-}$ mice (Figures 5C, 5D, and S4B). These data replicate those for the WT hFcγRIIB receptor (Figure 1F) and demonstrate that overcoming hFcγRIIB-mediated inhibition of depletion on effector cells is only efficiently achieved using an Fc-null hFcγRIIB-blocking mAb.

Previously, we hypothesized that the inability of a WT FcγRIIB blocking mAb to enhance target cell depletion was due to its Fc-mediated inhibitory signaling in myeloid effector cells (Figure 1G); however, in the NoTIM mouse, this is not possible due to the mutated ITIM. In the absence of a possible signaling effect and in light of the observation that inhibition correlates with surface expression, we considered whether hFcγRIIB impaired depletion by binding and sequestering the Fc of the opsonizing mAb, preventing engagement of activating FcγR. To examine this, the availability of activating mFcγRs on myeloid effector cells was analyzed *ex vivo* after mAb treatment. Treatment of NoTIM mice with an isotype control or rituximab did not alter mFcγR detection. In contrast, blockade with 6G (monotherapy or in combination with rituximab) greatly reduced the detection of mFcγRIV on splenic macrophages, with small decreases also in mFcγRI and mFcγRIII (Figures 5E and 5F). The effect was not observed with 6G-Q. Similar reductions in activating FcγR detection following 6G, but not 6G-Q, treatment was also observed on other potential effector cells (Ly6G$^+$ Ly6C$^+$ neutrophils and Ly6C^{hi} monocytes; Figures S4C and S4D). Similar experiments in hFcγRIIB Tg mice showed the same effect (Figure 5G). These data indicate the WT Fc region of 6G occupies the binding site of activating mFcγRs on effector cells, reducing the ability of the rituximab Fc to engage these same activating mFcγRs, decreasing target cell depletion. In contrast, the Fc-null 6G-Q does not block activating mFcγRs, leaving them to interact with the Fc of rituximab and elicit strong depletion (Figure S4B). Together, these experiments demonstrate that the cell surface expression of hFcγRIIB on myeloid effector cells is responsible for the suppression of anti-CD20 mAb-mediated target cell depletion through a signaling-independent mechanism, involving competition for Fc, and preventing engagement of activating mFcγRs.

Targeting of receptors concurrently on target cells and myeloid effectors impairs mAb-mediated depletion *in vivo*

We next considered whether this effect was related solely to targeting FcγRs or to the Fc-mediated blockade of activating FcγRs. To explore this, we performed experiments targeting hCD40, again when the antigen was on the target alone (B cells) (Figure S5A) or on both target and effector cells (Figure S5B). To achieve this, we transferred splenocytes from hCD40 Tg mice (T) alongside WT splenocytes (NT) into WT C57BL/6J or hCD40 Tg mice, treating them with effective depleting isotypes (mIgG2a or hIgG1) of anti-hCD40 mAb. Depletion of B cells was robust in WT mice (where the myeloid effector cells lack hCD40) but inefficient in hCD40 Tg recipients (Figure S5C). This was not due to the larger antigenic sink in the hCD40 Tg mice, as the serum levels of available mAb was indistinguishable in both (Figure S5D). To explore the underpinning mechanism, we again examined the availability of activating mFcγRs on myeloid effector cells. As before, we observed that, in instances where depletion is blunted (i.e., hCD40 Tg recipients), the staining of mFcγRs, including FcγRIV, was reduced (Figure S5E), but this was not the case in WT C57BL/6J recipients (Figure S5F).

hFcγRIIB impairs depletion of regulatory T cells in a signaling-independent manner

Having made these observations with B cell targets, we sought to evaluate other clinically relevant cellular targets, namely Tregs. Tregs regulate multiple facets of the immune response and their depletion is an important goal in several immunotherapy strategies (Elpek et al., 2007; Golgher et al., 2002; Onizuka et al., 1999; Rech et al., 2012) with CD25 a clinically relevant Treg target (Rech et al., 2012; Arce Vargas et al., 2017; Solomon et al., 2020). Accordingly, mFcγRII$^{-/-}$, hFcγRIIB Tg, and NoTIM mice were treated with anti-mCD25 mAb (clone: PC61) or isotype control and then the kinetics of CD4$^+$FOXP3$^+$ Treg depletion evaluated (Figure 6A). In agreement with the anti-mCD20 experiments, NoTIM mice were consistently more resistant to Treg depletion than both hFcγRIIB Tg and mFcγRII$^{-/-}$ mice, both from the blood and the spleen (Figure 6B). These data demonstrate an additional target and cell type where mAb-mediated depletion is impaired by hFcγRIIB in an ITIM signaling-independent manner.

hFcγRIIB impairs depletion of cells in a signaling-independent manner with impact on cancer immunotherapy

Next, we considered whether the impaired depletion of these various target cells in the NoTIM mouse would also occur in the tumor microenvironment (TME). To address this, we considered a fourth cell surface target receptor, OX40. Targeting mOX40 can deplete intratumoral Tregs via activating mFcγRs, leading to anti-tumor efficacy (Bulliard et al., 2014) and we

CellPress
OPEN ACCESS

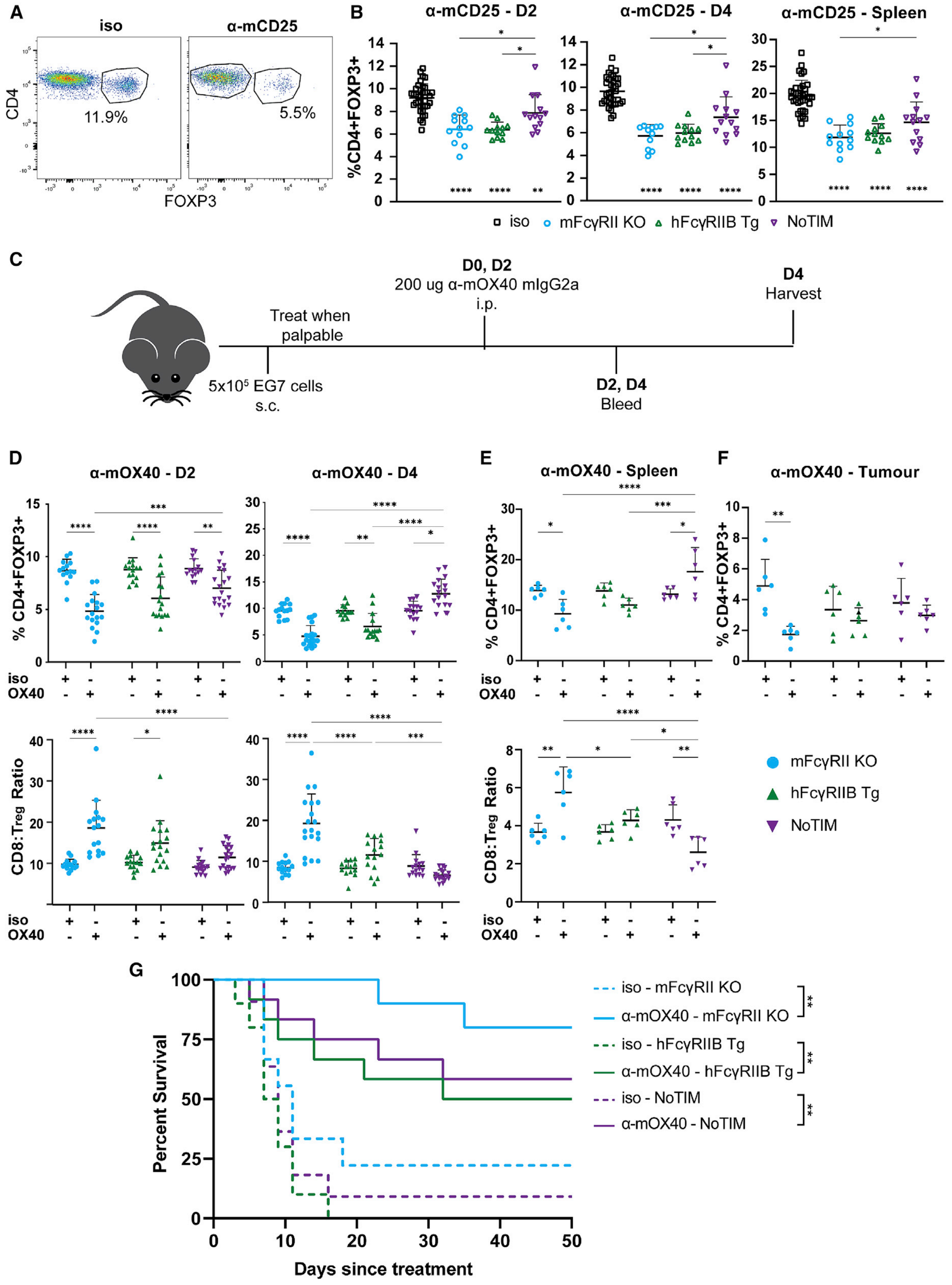

(legend on next page)

recently made similar observations for hOX40 (Griffiths et al., 2020). We therefore assessed the ability of the anti-mOX40 mAb OX86 mIgG2a to deplete Tregs in the EG7 thymoma model (Figure 6C). As with anti-mCD25, anti-mOX40 mAb depleted Treg efficiently from the blood (Figure 6D) and spleen (Figure 6E) of FcγRII$^{-/-}$ mice. hFcγRIIB Tg mice displayed intermediate depletion levels and NoTIM mice were most resistant, also reflected in the CD8:Treg ratios (Figures 6D and 6E lower panels). Treg depletion from the tumor replicated findings in the blood (Figure 6F). In accordance with earlier data (Bulliard et al., 2014), the reduced Treg depletion was associated with less effective tumor control in hFcγRIIB Tg and NoTIM mice, albeit without statistical significance (Figure 6G). We reasoned that the relative lack of therapeutic impact in the face of clear differences in Treg deletion was due to the ability of the anti-mOX40 mAb (like anti-4-1BB mAb) to evoke anti-tumor effects through multiple mechanisms. We have previously shown that mAb to these targets can elicit anti-tumor responses through either depletion of Tregs (requiring efficient engagement of activating mFcγRs) or direct co-stimulation on effector T cells (enhanced by inhibitory mFcγRII crosslinking) (Buchan et al., 2018; Griffiths et al., 2020), with the therapeutic impact the net result of these activities.

Therefore, we returned to a *bone fide* direct targeting mAb in a malignant B cell model lacking these complexities. We chose the Eμ-TCL1 lymphoma model (Bichi et al., 2002), previously used to assess depletion capabilities of anti-mCD20 mAb (Carter et al., 2017; Oldham et al., 2020; Tipton et al., 2015b). By inoculating mice with mCD20-expressing Eμ-TCL1 lymphoma cells, we were able to evaluate the concurrent anti-mCD20 mAb-mediated depletion of malignant and normal B cells from the blood as well as monitor tumor control (Figures 7A and 7B). Following tumor engraftment and detection in the blood, groups were randomized to receive anti-mCD20 mAb mIgG2a or isotype control and bled until the experimental endpoint was reached. Tumor cells were identified as CD19^{+}CD5^{+}B220^{lo} by FCM, alongside normal B cells (CD5^{-}B220^{hi}). Relative depletion was assessed after 2 weeks (Figure 7C) versus leukemia burden over time, in addition to long-term survival (Figures 7D and 7E). Growth of the Eμ-TCL1 cells in mFcγRII$^{-/-}$, hFcγRIIB Tg, and NoTIM mice was equivalent following treatment with the isotype control, whereas significant depletion resistance was observed in the NoTIM and to a lesser extent hFcγRIIB Tg mice (Figures 7C and 7D). Resistance was translated to a more rapid repopulation and expansion of the tumor cells after treatment, resulting in a shorter median time of survival (42 days for mFcγRII$^{-/-}$ versus 28 days for NoTIM mice; Figure 7E). Normal B cells also recovered much more rapidly in NoTIM versus mFcγRII$^{-/-}$ mice (Figure S6). Together, these data demonstrate that a non-signaling hFcγRIIB receptor (NoTIM) can strongly impair target cell depletion and compromise therapeutic efficacy.

DISCUSSION

Therapeutic mAbs remain the most exciting class of drugs, with >100 now approved (Kaplon et al., 2022). Although checkpoint blocking and immune-stimulatory mAbs are currently receiving much attention, >40% of approved therapeutic mAbs function through direct targeting of diseased cells, such as malignant or autoimmune B cells in the case of rituximab, and breast or colorectal cancer cells in the case of trastuzumab and cetuximab. Therapeutic efficacy relies at least in part on activating FcγRs and is impaired through expression of the inhibitory FcγR, FcγRIIB (Clynes et al., 2000). We previously showed blockade of hFcγRIIB using highly specific mAb enhanced anti-CD20 mAb-mediated depletion of both normal and malignant B cells *in vivo* (Roghanian et al., 2015) with the resulting combination currently being explored in the clinic (ClinicalTrials.gov registration numbers: NCT03571568; NCT04219254).

In the current study, we explored the properties of hFcγRIIB that impair target cell depletion and which format of mAb-mediated hFcγRIIB blockade was most effective to overcome them. We demonstrate that a WT Fc-functional anti-hFcγRIIB mAb provides optimal effects in systems where hFcγRIIB is expressed solely on the target (e.g., hFcγRIIB^{+} B cells), both in monotherapy and combination with hCD20 mAb. Similar effects were also shown recently by Lu et al. (2020), with the Fc-null (N297A) format failing to elicit direct cytotoxicity *in vitro* or efficacy *in vivo* in a variety of xenograft models. In contrast Fc-enhanced formats demonstrated more potent effects. However, it is important to note that these mAbs were explored in contexts that lacked hFcγRIIB expression on the host effector cells. In the current study, we also assessed the impact of blocking hFcγRIIB when hFcγRIIB was present on the effector cells but lacking from the target (hFcγRIIB^{-} B cells

Figure 6. Antibody-mediated depletion of regulatory T cells is impaired in NoTIM mice

(A) mFcγRII$^{-/-}$, hFcγRIIB Tg, or NoTIM mice were treated with PC61 rIgG1 i.p. on D0 and bled at D2 and D4 to analyze the kinetics of Treg depletion with the spleen assessed on D7. Example flow cytometry plots are shown; Tregs identified based on expression of CD4 and FOXP3.

(B) Percentage of Tregs in each group compared with isotype treated plotted as percentage of CD4+FOXP3+ cells. Result of three independent experiments. Line represents the mean (+SD). Statistical analyses: one-way ANOVA with Tukey's multiple comparisons. Significance under the data plots is treatment group compared with isotype. Significance above the data plots represents significance between treatment groups.

(C and D) Mice were injected with EG7 cells, then, once tumors were established, treated with OX86 mIgG2a or isotype control i.p. and then again 2 days later (C). Changes in Tregs are represented by percentage of CD4+FOXP3+ and CD8:Treg ratio (D). Result of three independent experiments. Statistical analyses: two-way ANOVA with Tukey's multiple comparisons.

(E and F) The same changes were assessed in spleen (E) and tumor (F) on D4. Result of two independent experiments. Line represents the mean (+SD). Statistical analyses: one-way ANOVA with Tukey's multiple comparisons. (F) Change in percentage of CD4+FOXP3+ on D4 in the tumor. Result of two independent experiments combined. Line represents the mean (+SD). Statistical analyses: one-way ANOVA with Tukey's multiple comparisons.

(G) Kaplan-Meier survival in EG7 tumor-bearing mice treated with OX86 mAb or isotype control. Statistical analyses: Log rank test. Statistical analyses next to isotype denotes significance between isotype and OX86-treated mice (e.g., NoTIM isotype and NoTIM treated). *p ≤ 0.05, **p ≤ 0.01, ***p ≤ 0.001, ****p ≤ 0.0001.

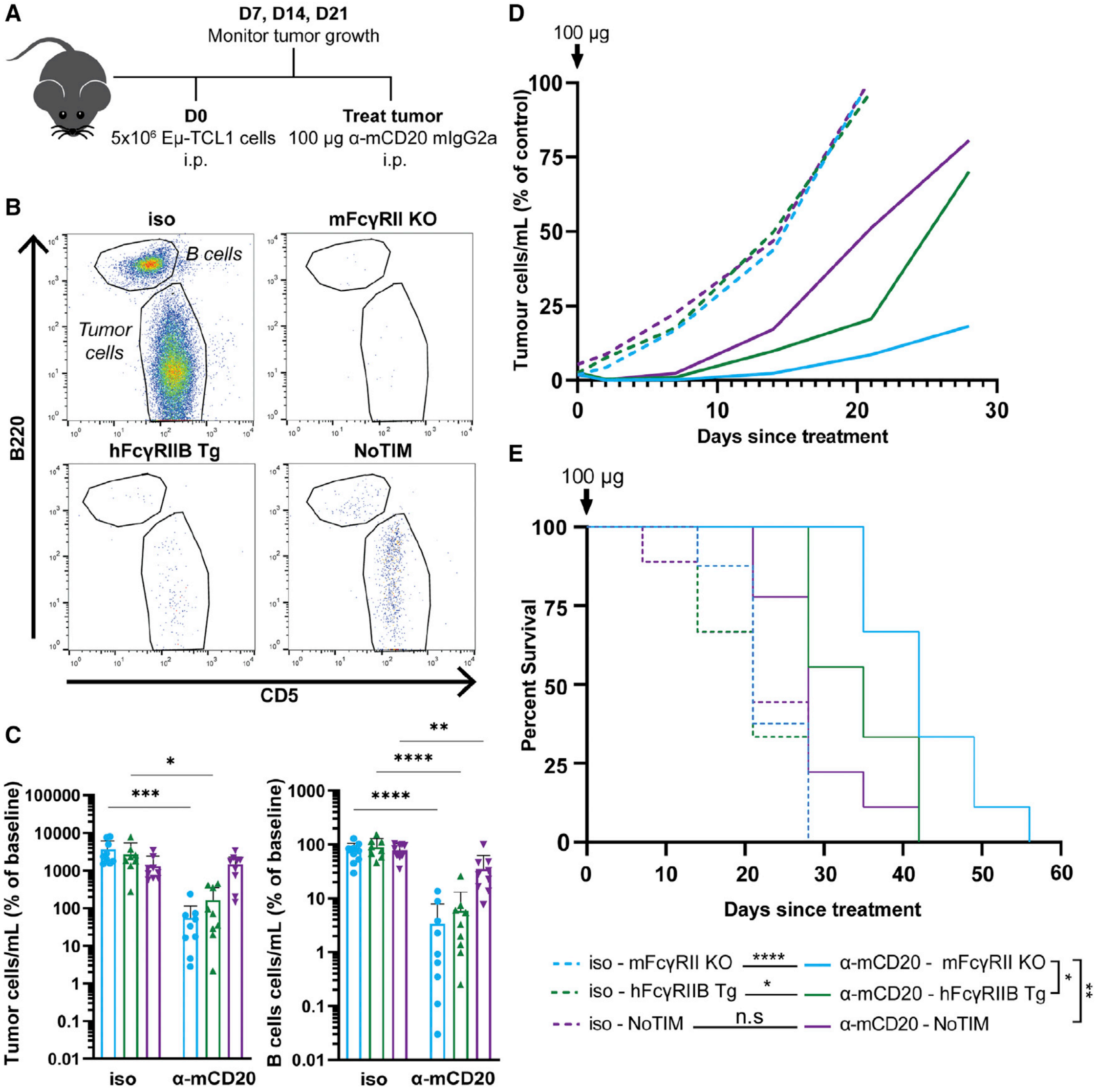

Figure 7. mAb-Antibody mediated depletion of malignant B cells is impaired in NoTIM mice, compromising therapy

(A) mFcγRII$^{-/-}$, hFcγRIIB Tg, or NoTIM mice were injected with Eμ-TCL1 cells and treated with 18B12 mIgG2a or isotype control as indicated.

(B) FCM was used to assess depletion of tumor (CD19+/B220^{lo}/CD5+) and normal B cells (CD19+/B200^{hi}/CD5−); plots show depletion after 2 days.

(C) Tumor cells and B cells were quantified on D14 and normalized to the number of cells before treatment to plot percentage change from baseline. Line shows the mean. Result of three independent experiments (n = 8–9 mice/group). Statistical analysis: one-way ANOVA with Tukey's multiple comparison test.

(D) The change in the proportion of tumor cells within the periphery following treatment were quantified by FCM over time.

(E) Survival analyzed using Kaplan-Meier. Statistical analysis: Log rank. Significance between isotype and 18B12-treated mice is denoted by the line between groups, while significance between treatment groups is defined by parenthesis between those treated with 18B12. *$p \leq 0.05$, **$p \leq 0.01$, ***$p \leq 0.001$, ****$p \leq 0.0001$.

or Tregs), reflective of most tumors outside of B cell malignancies. Here, an Fc-null hFcγRIIB mAb was most effective at augmenting target cell depletion in combination with a second therapeutic mAb, with Fc-functional anti-hFcγRIIB detrimental. In dissecting the basis for the preference for functional WT or Fc-null domains, we showed that hFcγRIIB ITIM phosphorylation was triggered by the former but not the latter in myeloid effector cells, initially supporting that hFcγRIIB mediates its inhibitory effects by ameliorating downstream signaling (Bruhns and Jonsson, 2015). However, our subsequent studies showed this not to be the case.

Employing a non-signaling, ITIM-mutated hFcγRIIB Tg (NoTIM) mouse model, we showed that ITIM signaling was dispensable for hFcγRIIB-mediated inhibition of target cell depletion. Strikingly, when mCD20 mAbs were administered *in vivo*, the NoTIM mice were more resistant to peripheral and tissue-resident B cell depletion than signaling-competent hFcγRIIB Tg or WT C57BL/6J mice. Resistance to depletion

was seen with both moderate (mIgG1) and strong (mIgG2a) depleting isotypes (Nimmerjahn and Ravetch, 2005). Importantly, hFcγRIIB-mediated internalization of the anti-mCD20 mAb from the target B cell surface was not different between cells expressing mFcγRII or hFcγRIIB. Additionally, the maintenance of the anti-mCD20 mAb in the serum was similar; indicating that neither activity was responsible for the resistance seen in the NoTIM mice. This was expected, given our previous demonstration that mAb:CD20:hFcγRIIB internalization appears linked to physical distortion of the plasma membrane, independent of hFcγRIIB signaling (Vaughan et al., 2015). Nevertheless, the relatively low level of internalization of anti-mCD20 mAb on both WT B cells and those expressing hFcγRIIB was initially surprising, given the rapid internalization of rituximab and hCD20 on normal B cells and B cell lymphomas (Lim et al., 2011; Vaughan et al., 2014). This low level of internalization is, however, fully in keeping with that seen with type II anti-CD20 mAbs such as obinutuzumab and indicates that 18B12 is a type II mAb (Lim et al., 2011; Vaughan et al., 2014) (further supported by our preliminary data showing that it is not effectively redistributed into lipid rafts [data not shown]). Using adoptive transfer models, we showed that expression of a non-signaling hFcγRIIB on effector cells, not target B cells, was responsible for the inhibition of mAb depletion. Inhibition was most likely achieved through competition between activating and inhibitory FcγRs for therapeutic mAb Fc. Moreover, anti-mCD25 and -mOX40 mAb-mediated depletion of Tregs highlighted that this phenomenon is not CD20 or B cell specific. Treg depletion was also blunted in the NoTIM compared with mFcγRII$^{-/-}$ mice and more prominent than in hFcγRIIB Tg mice. Therefore, hFcγRIIB negatively regulates direct targeting mAb in an ITIM signaling-independent manner.

We also explored means through which to optimally overcome hFcγRIIB. When target cells expressing the NoTIM receptor were transferred into mFcγRII$^{-/-}$ mice, an Fc-null anti-hFcγRIIB mAb was shown to have no inherent depleting activity and did not significantly improve rituximab-mediated depletion. In contrast, an Fc-WT anti-hFcγRIIB mAb induced potent depletion as monotherapy and alongside rituximab, demonstrating that ITIM signaling is not required when hFcγRIIB is operating as a B cell target and that targeting hFcγRIIB per se is not an issue for effective depletion.

In parallel experiments where the NoTIM was present on the effectors and not target cells, an Fc-functional hFcγRIIB-blocking mAb did not improve hCD20 mAb-mediated depletion in the NoTIM mice, whereas an Fc-null hFcγRIIB-blocking mAb did, providing depletion equivalent to that in mFcγRII$^{-/-}$ mice. In the absence of alternative explanations (accelerated loss of mAb from the serum, removal from the cell surface, or retained residual inhibitory signaling capacity) and the knowledge that surface presence correlated with activity, we explored receptor competition as the underlying mechanism. Occupancy data in the presence and absence of WT or Fc-null anti-hFcγRIIB mAb showed that the WT Fc was able to occupy the Fc-binding region of activating mFcγRs—namely, mFcγRIV with a significant effect seen on macrophages—reducing rituximab-FcγR interactions and depletion. mFcγRIV (along with mFcγRI) has been shown to be critical for mediating depletion of target cells (Gul et al., 2014); therefore, the significant effect seen on mFcγRIV in our data explains how WT hFcγRIIB mAb could reduce target cell depletion. mFcγR occupancy levels confirmed that the Fc-null hFcγRIIB mAb efficiently blocked hFcγRIIB without interaction with activating mFcγRs, increasing the probability of anti-hCD20 mAb Fc interacting with activating mFcγR and evoking depletion. This behavior has been reported previously, variously termed the Scorpion or Kurlander effect (Kurlander, 1980, 1983), and was described by us and others in examining expression and blockade of FcγR (Hamaguchi et al., 2006; Tipton et al., 2015a). Our experiments targeting hCD40 when expressed solely on the target cells or concurrently on the effector cells indicate the broad generality of our findings, demonstrating this same competition for FcγRs can result in thwarted target cell depletion when targeting antigens other than CD20 and hFcγRIIB.

To understand if this finding applied to other targets and cell types, and in a therapeutic context, anti-mCD25- and anti-mOX40-mediated depletion of Tregs was assessed. As before, NoTIM mice were resistant to mAb-mediated depletion of target cells. To target mCD25, we used PC61 rIgG1 that binds mFcγRIII as an activating FcγR (Arce Vargas et al., 2017), suggesting competition between hFcγRIIB and mFcγRIII for the Fc. This agrees with our data from targeting CD20, which show that competition between activating and inhibitory FcγRs drives reduced target cell depletion. They are also concordant with evidence that removal of mFcγRII$^{-/-}$ or selection of mAb isotypes with higher A:I ratio elicit greater Treg depletion (Arce Vargas et al., 2017). For targeting mOX40, we employed the more potent mIgG2a isotype of anti-mOX40 and showed similar inhibition of Treg depletion in the NoTIM mice. Furthermore, we were able to demonstrate that the lack of depletion rendered the mice more resistant to anti-OX40 mAb therapy of the EG7 thymoma. Finally, we returned to assess the ability of anti-mCD20 mAb to deplete malignant B cells in the various mouse strains, showing that, once again, expression of the NoTIM receptor impaired target cell depletion, in this case of Eμ-TCL1 tumor cells. Less effective depletion led to more rapid tumor repopulation and shorter median survival compared with mice lacking the receptor.

These results were initially surprising as a substantial body of work suggests ITIM signaling is central to the inhibitory activity of hFcγRIIB and other inhibitory receptors (Getahun and Cambier, 2015; Muta et al., 1994; Stopforth et al., 2016). However, when hFcγRIIB is assessed in relation to other ITIM-containing immunoreceptors, it seems unlikely to deliver its regulatory function through ITIM signaling alone. hFcγRIIB possesses a single ITIM, whereas most inhibitory receptors contain two or more, with inhibitory leukocyte Ig-like receptors (LILR) ranging from two to four ITIMs (De Louche and Roghanian, 2022; van der Touw et al., 2017), SIRPα three ITIMs (Zen et al., 2013), and LAIR1 two ITIMs (Kang et al., 2015). Furthermore, there are usually multiple inhibitory receptors capable of regulating multiple activating receptors, such as within the KIR receptor system (Rajalingam, 2012). In addition, ITIM-containing inhibitory receptors largely bind to ligands without competition (e.g., SIRPα and CD47) or through interactions between activating and inhibitory receptors that have very different ligands (KIR and LILR families),

allowing for effective transduction of inhibitory regulation. In contrast, hFcγRIIB, as the sole inhibitory IgG receptor of the FcγR family and with a single ITIM, does not have any of the additional advantages of other inhibitory receptors that allow potent regulatory signaling. Taken together, it seems unlikely that hFcγRIIB ITIM-mediated signaling would be able to singularly regulate the diverse activities of multiple ITAM-containing activating FcγRs. In this context, additional regulation through competition for Fc, as described here, would afford greater inhibitory capacity. Although not studied here, and not relevant for the effector cells, the ITIM-containing Fc receptor-like (FCRL) family of receptors, such as FCRL2-6, may contribute more widely to Fc-mediated outcomes on B cells in humans (Li et al., 2014). It is also worth noting that alternative means of inhibiting activating FcγRs exist, such as the ITAMi pathway, evoked following sub-optimal receptor engagement (Aloulou et al., 2012).

Overall, our study demonstrates that inhibition of target cell depletion by direct targeting therapeutic mAb is mediated by hFcγRIIB on effector cells through competition between activating FcγRs and hFcγRIIB for the Fc of the therapeutic mAb in an ITIM signaling-independent manner. This inhibition of myeloid effector cells can be overcome through use of an appropriately engineered (Fc-null) anti-hFcγRIIB mAb, restoring engagement of activating FcγRs of the Fc of the therapeutic mAb.

Limitations of the study

One limitation of our study was that it employs mice transgenic for just a single human FcγR, FcγRIIB. It therefore evaluates the impact of hFcγRIIB on murine activating FcγR, not human activating FcγR. Although both species exhibit broadly equivalent systems of FcγR-mediated depletion, it remains possible that differences exist. In addition, the expression level of FcγRIIB was not identical in all hFcγRIIB Tg and NoTIM mice. Although the variable level of hFcγRIIB expression in the hFcγRIIB Tg mice was useful in helping demonstrate expression:depletion relationships (Figure S2), it would have been ideal to have equivalent expression in both strains.

STAR★METHODS

Detailed methods are provided in the online version of this paper and include the following:

- KEY RESOURCES TABLE
- RESOURCE AVAILABILITY
  - Lead contact
  - Materials availability
  - Data and code availability
- EXPERIMENTAL MODEL AND SUBJECT DETAILS
  - Mice
  - Human samples
  - Cell lines
- METHOD DETAILS
  - Antibodies and reagents
  - Flow cytometry (FCM)
  - Immunofluorescence
  - Cell isolation
  - Generation of mouse bone marrow-derived macrophages (BMDM)
  - mCD20 mIgG1 internalization assay
  - Calcium flux assay
  - Preparation of heat aggregated human IgG
  - Heat aggregated human IgG internalization assay
  - Western Blotting
  - Cell binding assay
  - Adoptive B cell transfer assay
  - *In vivo* Raji xenograft therapy
  - CLL patient derived xenograft model
  - *In vivo* B cell depletion
  - *In vivo* regulatory T cell depletion using PC61
  - *In vivo* regulatory T cell depletion using OX86 in E.G7 tumor bearing mice
  - *In vivo* Eμ-TCL1 lymphoma depletion
- QUANTIFICATION AND STATISTICAL ANALYSIS

SUPPLEMENTAL INFORMATION

Supplemental information can be found online at https://doi.org/10.1016/j.celrep.2022.111071.

ACKNOWLEDGMENTS

We thank members of the Antibody and Vaccine Group and BioInvent International for useful discussions and the pre-clinical unit staff for animal husbandry. Funding was from Blood Cancer UK (award numbers 12050 and 14043), CRUK (award numbers A18087, A24721, A25139), and BioInvent International. A.P.S was supported by an iCASE studentship from the BBSRC (1943869) in association with GSK.

AUTHOR CONTRIBUTIONS

A.P.S. designed and performed experiments, analyzed and interpreted data, and wrote the manuscript. A.R. designed and performed experiments, supervised data collection, analyzed and interpreted data, and edited the manuscript. R.O., H.T.C., K.L.C., Y.B., S.J., and L.N.D. designed and performed experiments. C.P., J.K., T.I., I.M., and A.T. generated or provided key reagents or performed and analyzed research. D.R. and P.M. helped design the study, supervised data collection, and discussed and interpreted data. I.T. helped design the study, supervised data collection, and discussed and interpreted data. B.F., S.A.B., and M.S.C. designed the study, supervised data collection, discussed and interpreted data, and wrote the manuscript.

DECLARATION OF INTERESTS

M.S.C. acts as a consultant for a number of biotech companies, being retained as a consultant for BioInvent International, and has received research funding from BioInvent International, GSK, UCB, iTeos, and Roche. S.A.B. has acted as a consultant for a number of biotechs and receives institutional support for grants and patents from BioInvent. A.R. receives institutional support for grants and patents from BioInvent International. D.R. and P.M. are employees of GSK. I.T. and B.F. are employees of BioInvent International. This work is related to patent family EP3263602A1 concerning the combined use of FcγRIIB and CD20-specific antibodies as well as US 11001638 and WO2019020774-A2/A3, relating to OX40 and 4-1BB.

Received: February 20, 2022
Revised: May 10, 2022
Accepted: June 23, 2022
Published: July 19, 2022

REFERENCES

Ahuja, A., Shupe, J., Dunn, R., Kashgarian, M., Kehry, M.R., and Shlomchik, M.J. (2007). Depletion of B cells in murine lupus: efficacy and resistance. J. Immunol. *179*, 3351–3361. https://doi.org/10.4049/jimmunol.179.5.3351.

Aloulou, M., Ben Mkaddem, S., Biarnes-Pelicot, M., Boussetta, T., Souchet, H., Rossato, E., Benhamou, M., Crestani, B., Zhu, Z., Blank, U., et al. (2012). IgG1 and IVIg induce inhibitory ITAM signaling through FcγRIII controlling inflammatory responses. Blood *119*, 3084–3096. https://doi.org/10.1182/blood-2011-08-376046.

Arce Vargas, F., Furness, A.J., Solomon, I., Joshi, K., Mekkaoui, L., Lesko, M.H., Miranda Rota, E., Dahan, R., Georgiou, A., Sledzinska, A., et al. (2017). Fc-optimized anti-CD25 depletes tumor-infiltrating regulatory T cells and synergizes with PD-1 blockade to eradicate established tumors. Immunity *46*, 577–586. https://doi.org/10.1016/j.immuni.2017.03.013.

Barrington, R.A., Pozdnyakova, O., Zafari, M.R., Benjamin, C.D., and Carroll, M.C. (2002). B lymphocyte memory: role of stromal cell complement and FcgammaRIIB receptors. J. Exp. Med. *196*, 1189–1200. https://doi.org/10.1084/jem.20021110.

Beers, S.A., Chan, C.H.T., James, S., French, R.R., Attfield, K.E., Brennan, C.M., Ahuja, A., Shlomchik, M.J., Cragg, M.S., and Glennie, M.J. (2008). Type II (tositumomab) anti-CD20 monoclonal antibody out performs type I (rituximab-like) reagents in B-cell depletion regardless of complement activation. Blood *112*, 4170–4177. https://doi.org/10.1182/blood-2008-04-149161.

Beers, S.A., French, R.R., Chan, H.T.C., Lim, S.H., Jarrett, T.C., Vidal, R.M., Wijayaweera, S.S., Dixon, S.V., Kim, H., Cox, K.L., et al. (2010). Antigenic modulation limits the efficacy of anti-CD20 antibodies: implications for antibody selection. Blood *115*, 5191–5201. https://doi.org/10.1182/blood-2010-01-263533.

Bichi, R., Shinton, S.A., Martin, E.S., Koval, A., Calin, G.A., Cesari, R., Russo, G., Hardy, R.R., and Croce, C.M. (2002). Human chronic lymphocytic leukemia modeled in mouse by targeted TCL1 expression. Proc. Natl. Acad. Sci. USA *99*, 6955–6960. https://doi.org/10.1073/pnas.102181599.

Bruhns, P. (2012). Properties of mouse and human IgG receptors and their contribution to disease models. Blood *119*, 5640–5649. https://doi.org/10.1182/blood-2012-01-380121.

Bruhns, P., and Jönsson, F. (2015). Mouse and human FcR effector functions. Immunol. Rev. *268*, 25–51. https://doi.org/10.1111/imr.12350.

Buchan, S.L., Dou, L., Remer, M., Booth, S.G., Dunn, S.N., Lai, C., Semmrich, M., Teige, I., Mårtensson, L., Penfold, C.A., et al. (2018). Antibodies to costimulatory receptor 4-1BB enhance anti-tumor immunity via T regulatory cell depletion and promotion of CD8 T cell effector function. Immunity *49*, 958–970.e7. https://doi.org/10.1016/j.immuni.2018.09.014.

Bulliard, Y., Jolicoeur, R., Zhang, J., Dranoff, G., Wilson, N.S., and Brogdon, J.L. (2014). OX40 engagement depletes intratumoral Tregs via activating FcγRs, leading to antitumor efficacy. Immunol. Cell Biol. *92*, 475–480. https://doi.org/10.1038/icb.2014.26.

Carter, M.J., Cox, K.L., Blakemore, S.J., Turaj, A.H., Oldham, R.J., Dahal, L.N., Tannheimer, S., Forconi, F., Packham, G., and Cragg, M.S. (2017). PI3Kδ inhibition elicits anti-leukemic effects through Bim-dependent apoptosis. Leukemia *31*, 1423–1433. https://doi.org/10.1038/leu.2016.333.

Chan, A.C., and Carter, P.J. (2010). Therapeutic antibodies for autoimmunity and inflammation. Nat. Rev. Immunol. *10*, 301–316. https://doi.org/10.1038/nri2761.

Clynes, R., Maizes, J.S., Guinamard, R., Ono, M., Takai, T., and Ravetch, J.V. (1999). Modulation of immune complex-induced inflammation in vivo by the coordinate expression of activation and inhibitory Fc receptors. J. Exp. Med. *189*, 179–186. https://doi.org/10.1084/jem.189.1.179.

Clynes, R.A., Towers, T.L., Presta, L.G., and Ravetch, J.V. (2000). Inhibitory Fc receptors modulate in vivo cytoxicity against tumor targets. Nat. Med. *6*, 443–446. https://doi.org/10.1038/74704.

Daëron, M., Latour, S., Malbec, O., Espinosa, E., Pina, P., Pasmans, S., and Fridman, W.H. (1995). The same tyrosine-based inhibition motif, in the intracytoplasmic domain of Fc gamma RIIB, regulates negatively BCR-TCR-and FcR-dependent cell activation. Immunity *3*, 635–646. https://doi.org/10.1016/1074-7613(95)90134-5.

de Haij, S., Jansen, J.M., Boross, P., Beurskens, F.J., Bakema, J.E., Bos, D.L., Martens, A., Verbeek, J.S., Parren, P.W., van de Winkel, J.G., and Leusen, J.H. (2010). In vivo cytotoxicity of type I CD20 antibodies critically depends on Fc receptor ITAM signaling. Cancer Res. *70*, 3209–3217. https://doi.org/10.1158/0008-5472.CAN-09-4109.

De Louche, C.D., and Roghanian, A. (2022). Human inhibitory leukocyte Ig-like receptors: from immunotolerance to immunotherapy. JCI Insight *7*, e151553. https://doi.org/10.1172/jci.insight.151553.

DiLillo, D.J., and Ravetch, J.V. (2015). Fc-receptor interactions regulate both cytotoxic and immunomodulatory therapeutic antibody effector functions. Cancer Immunol Res *3*, 704–713. https://doi.org/10.1158/2326-6066.CIR-15-0120.

Elpek, K.G., Lacelle, C., Singh, N.P., Yolcu, E.S., and Shirwan, H. (2007). CD4+CD25+ T regulatory cells dominate multiple immune evasion mechanisms in early but not late phases of tumor development in a B cell lymphoma model. J. Immunol. *178*, 6840–6848. https://doi.org/10.4049/jimmunol.178.11.6840.

Ganesan, L.P., Kim, J., Wu, Y., Mohanty, S., Phillips, G.S., Birmingham, D.J., Robinson, J.M., and Anderson, C.L. (2012). FcγRIIb on liver sinusoidal endothelium clears small immune complexes. J. Immunol. *189*, 4981–4988. https://doi.org/10.4049/jimmunol.1202017.

Getahun, A., and Cambier, J.C. (2015). Of ITIMs, ITAMs, and ITAMis: revisiting immunoglobulin Fc receptor signaling. Immunol. Rev. *268*, 66–73. https://doi.org/10.1111/imr.12336.

Glennie, M.J., French, R.R., Cragg, M.S., and Taylor, R.P. (2007). Mechanisms of killing by anti-CD20 monoclonal antibodies. Mol. Immunol. *44*, 3823–3837. https://doi.org/10.1016/j.molimm.2007.06.151.

Golgher, D., Jones, E., Powrie, F., Elliott, T., and Gallimore, A. (2002). Depletion of CD25+ regulatory cells uncovers immune responses to shared murine tumor rejection antigens. Eur. J. Immunol. *32*, 3267–3275. https://doi.org/10.1002/1521-4141(200211)32:11<3267::AID-IMMU3267>3.0.CO;2-1.

Grandjean, C.L., Montalvao, F., Celli, S., Michonneau, D., Breart, B., Garcia, Z., Perro, M., Freytag, O., Gerdes, C.A., and Bousso, P. (2016). Intravital imaging reveals improved Kupffer cell-mediated phagocytosis as a mode of action of glycoengineered anti-CD20 antibodies. Sci. Rep. *6*, 34382. https://doi.org/10.1038/srep34382.

Griffiths, J., Hussain, K., Smith, H.L., Sanders, T., Cox, K.L., Semmrich, M., Mårtensson, L., Kim, J., Inzhelevskaya, T., Penfold, C.A., et al. (2020). Domain binding and isotype dictate the activity of anti-human OX40 antibodies. J Immunother Cancer *8*, e001557. https://doi.org/10.1136/jitc-2020-001557.

Gül, N., Babes, L., Siegmund, K., Korthouwer, R., Bögels, M., Braster, R., Vidarsson, G., ten Hagen, T.L., Kubes, P., and van Egmond, M. (2014). Macrophages eliminate circulating tumor cells after monoclonal antibody therapy. J. Clin. Invest. *124*, 812–823. https://doi.org/10.1172/JCI66776.

Hamaguchi, Y., Xiu, Y., Komura, K., Nimmerjahn, F., and Tedder, T.F. (2006). Antibody isotype-specific engagement of Fcγ receptors regulates B lymphocyte depletion during CD20 immunotherapy. J. Exp. Med. *203*, 743–753. https://doi.org/10.1084/jem.20052283.

Huang, Z.Y., Hunter, S., Kim, M.K., Indik, Z.K., and Schreiber, A.D. (2003). The effect of phosphatases SHP-1 and SHIP-1 on signaling by the ITIM- and ITAM-containing Fcγ receptors FcγRIIB and FcγRIIA. J. Leukoc. Biol. *73*, 823–829. https://doi.org/10.1189/jlb.0902454.

Illidge, T., Honeychurch, J., Howatt, W., Ross, F., Wilkins, B., and Crags, M. (2000). A new in vivo and in vitro B cell lymphoma model, pi-BCL1. Cancer Biother. Radiopharm. *15*, 571–580. https://doi.org/10.1089/cbr.2000.15.571.

Kang, X., Lu, Z., Cui, C., Deng, M., Fan, Y., Dong, B., Han, X., Xie, F., Tyner, J.W., Coligan, J.E., et al. (2015). The ITIM-containing receptor LAIR1 is essential for acute myeloid leukaemia development. Nat. Cell Biol. *17*, 665–677. https://doi.org/10.1038/ncb3158.

CellPress
OPEN ACCESS

Kaplon, H., Chenoweth, A., Crescioli, S., and Reichert, J.M. (2022). Antibodies to watch in 2022. mAbs *14*, 2014296. https://doi.org/10.1080/19420862.2021.2014296.

Koenderman, L. (2019). Inside-out control of Fc-receptors. Front. Immunol. *10*, 544. https://doi.org/10.3389/fimmu.2019.00544.

Kurlander, R.J. (1980). Reversible and irreversible loss of Fc receptor function of human monocytes as a consequence of interaction with immunoglobulin G. J. Clin. Invest. *66*, 773–781. https://doi.org/10.1172/JCI109915.

Kurlander, R.J. (1983). Blockade of Fc receptor-mediated binding to U-937 cells by murine monoclonal antibodies directed against a variety of surface antigens. J. Immunol. *131*, 140–147.

Lee, C.S., Ashton-Key, M., Cogliatti, S., Rondeau, S., Schmitz, S.F.H., Ghielmini, M., Cragg, M.S., and Johnson, P. (2015). Expression of the inhibitory Fc gamma receptor IIB (FCGR2B, CD32B) on follicular lymphoma cells lowers the response rate to rituximab monotherapy (SAKK 35/98). Br. J. Haematol. *168*, 145–148. https://doi.org/10.1111/bjh.13071.

Li, F., and Ravetch, J.V. (2013). Antitumor activities of agonistic anti-TNFR antibodies require differential FcγRIIB coengagement in vivo. Proc. Natl. Acad. Sci. USA *110*, 19501–19506. https://doi.org/10.1073/pnas.1319502110.

Li, F.J., Won, W.J., Becker, E.J., Jr., Easlick, J.L., Tabengwa, E.M., Li, R., Shakhmatov, M., Honjo, K., Burrows, P.D., and Davis, R.S. (2014). Emerging roles for the FCRL family members in lymphocyte biology and disease. Curr. Top. Microbiol. Immunol. *382*, 29–50. https://doi.org/10.1007/978-3-319-07911-0_2.

Lim, S.H., Vaughan, A.T., Ashton-Key, M., Williams, E.L., Dixon, S.V., Chan, H.T.C., Beers, S.A., French, R.R., Cox, K.L., Davies, A.J., et al. (2011). Fc gamma receptor IIb on target B cells promotes rituximab internalization and reduces clinical efficacy. Blood *118*, 2530–2540. https://doi.org/10.1182/blood-2011-01-330357.

Lu, H., Molony, R.D., Chen, D., Jang, S., Wolf, B., Ewert, S., Flaherty, M., Xu, F., Isim, S., Shim, Y., et al. (2020). Development of anti-CD32b antibodies with enhanced Fc function for the treatment of B and plasma cell malignancies. Mol. Cancer Therapeut. *19*, 2089–2104. https://doi.org/10.1158/1535-7163.MCT-19-0003.

Miettinen, H.M., Matter, K., Hunziker, W., Rose, J.K., and Mellman, I. (1992). Fc receptor endocytosis is controlled by a cytoplasmic domain determinant that actively prevents coated pit localization. J. Cell Biol. *116*, 875–888. https://doi.org/10.1083/jcb.116.4.875.

Muta, T., Kurosaki, T., Misulovin, Z., Sanchez, M., Nussenzweig, M.C., and Ravetch, J.V. (1994). A 13-amino-acid motif in the cytoplasmic domain of Fc gamma RIIB modulates B-cell receptor signalling. Nature *368*, 70–73. https://doi.org/10.1038/368070a0.

Nimmerjahn, F., and Ravetch, J.V. (2005). Divergent immunoglobulin g subclass activity through selective Fc receptor binding. Science *310*, 1510–1512. https://doi.org/10.1126/science.1118948.

Nowicka, M., Hilton, L.K., Ashton-Key, M., Hargreaves, C.E., Lee, C., Foxall, R., Carter, M.J., Beers, S.A., Potter, K.N., Bolen, C.R., et al. (2021). Prognostic significance of FCGR2B expression for the response of DLBCL patients to rituximab or obinutuzumab treatment. Blood Adv. *5*, 2945–2957. https://doi.org/10.1182/bloodadvances.2021004770.

Oldham, R.J., Mockridge, C.I., James, S., Duriez, P.J., Chan, H.T.C., Cox, K.L., Pitic, V.A., Glennie, M.J., and Cragg, M.S. (2020). FcγRII (CD32) modulates antibody clearance in NOD SCID mice leading to impaired antibody-mediated tumor cell deletion. J Immunother Cancer *8*, e000619. https://doi.org/10.1136/jitc-2020-000619.

Onizuka, S., Tawara, I., Shimizu, J., Sakaguchi, S., Fujita, T., and Nakayama, E. (1999). Tumor rejection by in vivo administration of anti-CD25 (interleukin-2 receptor alpha) monoclonal antibody. Cancer Res. *59*, 3128–3133.

Ono, M., Okada, H., Bolland, S., Yanagi, S., Kurosaki, T., and Ravetch, J.V. (1997). Deletion of SHIP or SHP-1 reveals two distinct pathways for inhibitory signaling. Cell *90*, 293–301. https://doi.org/10.1016/s0092-8674(00)80337-2.

Rajalingam, R. (2012). Overview of the killer cell immunoglobulin-like receptor system. Methods Mol. Biol. *882*, 391–414. https://doi.org/10.1007/978-1-61779-842-9_23.

Rech, A.J., Mick, R., Martin, S., Recio, A., Aqui, N.A., Powell, D.J., Jr., Colligon, T.A., Trosko, J.A., Leinbach, L.I., Pletcher, C.H., et al. (2012). CD25 blockade depletes and selectively reprograms regulatory T cells in concert with immunotherapy in cancer patients. Sci. Transl. Med. *4*, 134ra62. https://doi.org/10.1126/scitranslmed.3003330.

Roghanian, A., Cragg, M.S., and Frendéus, B. (2016). Resistance is futile: targeting the inhibitory FcγRIIB (CD32B) to maximize immunotherapy. OncoImmunology *5*, e1069939. https://doi.org/10.1080/2162402X.2015.1069939.

Roghanian, A., Hu, G., Fraser, C., Singh, M., Foxall, R.B., Meyer, M.J., Lees, E., Huet, H., Glennie, M.J., Beers, S.A., et al. (2019). Cyclophosphamide enhances cancer antibody immunotherapy in the resistant bone marrow niche by modulating macrophage FcγR expression. Cancer Immunol. Res. *7*, 1876–1890. https://doi.org/10.1158/2326-6066.CIR-18-0835.

Roghanian, A., Stopforth, R.J., Dahal, L.N., and Cragg, M.S. (2018). New revelations from an old receptor: immunoregulatory functions of the inhibitory Fc gamma receptor, FcγRIIB (CD32B). J. Leukoc. Biol. *103*, 1077–1088. https://doi.org/10.1002/JLB.2MIR0917-354R.

Roghanian, A., Teige, I., Mårtensson, L., Cox, K., Kovacek, M., Ljungars, A., Mattson, J., Sundberg, A., Vaughan, A., Shah, V., et al. (2015). Antagonistic human FcγRIIB (CD32B) antibodies have anti-tumor activity and overcome resistance to antibody therapy In Vivo. Cancer Cell *27*, 473–488. https://doi.org/10.1016/j.ccell.2015.03.005.

Scott, A.M., Wolchok, J.D., and Old, L.J. (2012). Antibody therapy of cancer. Nat. Rev. Cancer *12*, 278–287. https://doi.org/10.1038/nrc3236.

Setiady, Y.Y., Coccia, J.A., and Park, P.U. (2010). *In vivo*depletion of $CD4^+FOXP3^+$Treg cells by the PC61 anti-CD25 monoclonal antibody is mediated by $Fc\gamma RIII^+$phagocytes. Eur. J. Immunol. *40*, 780–786. https://doi.org/10.1002/eji.200939613.

Solomon, I., Amann, M., Goubier, A., Arce Vargas, F., Zervas, D., Qing, C., Henry, J.Y., Ghorani, E., Akarca, A.U., Marafioti, T., et al. (2020). CD25-Treg-depleting antibodies preserving IL-2 signaling on effector T cells enhance effector activation and antitumor immunity. Nat Cancer *1*, 1153–1166. https://doi.org/10.1038/s43018-020-00133-0.

Stopforth, R.J., Cleary, K.L.S., and Cragg, M.S. (2016). Regulation of monoclonal antibody immunotherapy by FcγRIIB. J. Clin. Immunol. *36*, 88–94. https://doi.org/10.1007/s10875-016-0247-8.

Stopforth, R.J., Oldham, R.J., Tutt, A.L., Duriez, P., Chan, H.T.C., Binkowski, B.F., Zimprich, C., Li, D., Hargreaves, P.G., Cong, M., et al. (2018). Detection of experimental and clinical immune complexes by measuring SHIP-1 recruitment to the inhibitory FcγRIIB. J. Immunol. *200*, 1937–1950. https://doi.org/10.4049/jimmunol.1700832.

Tipton, T.R.W., Mockridge, C.I., French, R.R., Tutt, A.L., Cragg, M.S., and Beers, S.A. (2015a). Anti-mouse FcγRIV antibody 9E9 also blocks FcγRIII in vivo. Blood *126*, 2643–2645. https://doi.org/10.1182/blood-2015-09-671339.

Tipton, T.R.W., Roghanian, A., Oldham, R.J., Carter, M.J., Cox, K.L., Mockridge, C.I., French, R.R., Dahal, L.N., Duriez, P.J., Hargreaves, P.G., et al. (2015b). Antigenic modulation limits the effector cell mechanisms employed by type I anti-CD20 monoclonal antibodies. Blood *125*, 1901–1909. https://doi.org/10.1182/blood-2014-07-588376.

van der Touw, W., Chen, H.M., Pan, P.Y., and Chen, S.H. (2017). LILRB receptor-mediated regulation of myeloid cell maturation and function. Cancer Immunol. Immunother. *66*, 1079–1087. https://doi.org/10.1007/s00262-017-2023-x.

Vaughan, A.T., Chan, C.H., Klein, C., Glennie, M.J., Beers, S.A., and Cragg, M.S. (2015). Activatory and inhibitory Fcγ receptors augment rituximab-mediated internalization of CD20 independent of signaling via the cytoplasmic domain. J. Biol. Chem. *290*, 5424–5437. https://doi.org/10.1074/jbc.M114.593806.

Vaughan, A.T., Iriyama, C., Beers, S.A., Chan, C.H.T., Lim, S.H., Williams, E.L., Shah, V., Roghanian, A., Frendéus, B., Glennie, M.J., and Cragg, M.S. (2014). Inhibitory FcγRIIb (CD32b) becomes activated by therapeutic mAb in both cis and trans and drives internalization according to antibody specificity. Blood *123*, 669–677. https://doi.org/10.1182/blood-2013-04-490821.

White, A., Chan, H., French, R., Willoughby, J., Mockridge, C., Roghanian, A., Penfold, C., Booth, S., Dodhy, A., Polak, M., et al. (2015). Conformation of the human immunoglobulin G2 hinge imparts superagonistic properties to immunostimulatory anticancer antibodies. Cancer Cell *27*, 138–148. https://doi.org/10.1016/j.ccell.2014.11.001.

White, A.L., Chan, H.T.C., Roghanian, A., French, R.R., Mockridge, C.I., Tutt, A.L., Dixon, S.V., Ajona, D., Verbeek, J.S., Al-Shamkhani, A., et al. (2011). Interaction with FcγRIIB is critical for the agonistic activity of anti-CD40 monoclonal antibody. J. Immunol. *187*, 1754–1763. https://doi.org/10.4049/jimmunol.1101135.

Williams, E.L., Tutt, A.L., Beers, S.A., French, R.R., Chan, C.H.T., Cox, K.L., Roghanian, A., Penfold, C.A., Butts, C.L., Boross, P., et al. (2013). Immunotherapy targeting inhibitory Fcγ receptor IIB (CD32b) in the mouse is limited by monoclonal antibody consumption and receptor internalization. J. Immunol. *191*, 4130–4140. https://doi.org/10.4049/jimmunol.1301430.

Xiang, Z., Cutler, A.J., Brownlie, R.J., Fairfax, K., Lawlor, K.E., Severinson, E., Walker, E.U., Manz, R.A., Tarlinton, D.M., and Smith, K.G.C. (2007). FcγRIIb controls bone marrow plasma cell persistence and apoptosis. Nat. Immunol. *8*, 419–429. https://doi.org/10.1038/ni1440.

Zen, K., Guo, Y., Bian, Z., Lv, Z., Zhu, D., Ohnishi, H., Matozaki, T., and Liu, Y. (2013). Inflammation-induced proteolytic processing of the SIRPα cytoplasmic ITIM in neutrophils propagates a proinflammatory state. Nat. Commun. *4*, 2436. https://doi.org/10.1038/ncomms3436.

Zhang, Y., Huo, M., Zhou, J., and Xie, S. (2010). PKSolver: an add-in program for pharmacokinetic and pharmacodynamic data analysis in Microsoft Excel. Comput. Methods Progr. Biomed. *99*, 306–314. https://doi.org/10.1016/j.cmpb.2010.01.007.

STAR★METHODS

KEY RESOURCES TABLE

REAGENT or RESOURCE	SOURCE	IDENTIFIER
Antibodies		
F(ab')$_2$ anti-human hFcγRII (Clone: AT10) (FITC)	In-house	N/A
hIgG1 anti-human hFcγRIIB (Clone: 6G11) (AlexaFluor 488)	BioInvent International AB	https://doi.org/10.1016/j.ccell.2015.03.005
mIgG1 anti-human hFcγRIII (Clone: 3G8) (PE)	In-house	N/A
hIgG1 anti-human CD20 (Clone: Rituximab) (AlexaFluor 488)	University Hospital Southampton Pharmacy	N/A
mIgG1 anti-human CD19 (Clone HIB19) (APC)	Biolegend	302212
mIgG2a anti-human CD14 (Clone: M5E2) (Pacific Blue)	Biolegend	301815
mIgG1 anti-human CD56 (Clone: 5.1H11) (APC/Cy7)	Biolegend	362512
F(ab')$_2$ anti-BCL-1 idiotype (Clone: MC106A5) (FITC)	In-house	N/A
F(ab')$_2$ anti-mouse FcγRI (Clone: AT 152-9) (FITC)	In-house	N/A
F(ab')$_2$ anti-mouse FcγRII (Clone: AT 130-2) (FITC)	In-house	N/A
F(ab')$_2$ anti-mouse FcγRIII (Clone: AT 154-2) (FITC)	In-house	N/A
hamster IgG anti-mouse FcγRIV (Clone: 9E9) (FITC)	In-house	N/A
mIgG1 anti-mouse CD20 (Clone: 18B12) (AlexaFluor 488)	In-house	N/A
rIgG2b N297A anti-mouse F4/80 (Clone: CI:A3-1) (AlexaFluor 647)	In-house	N/A
rIgG2b anti-mouse F4/80 (Clone: CI:A3-1) (APC)	BioRad	MCA497APC
rIgG2a anti-mouse CD19 (Clone: 1D3/CD19) (PE)	Biolegend	152408
rIgG2a anti-mouse/human B220 (Clone: RA3-6B2) (APC)	Biolegend	103212
rIgG2b anti-mouse CD11B (Clone: M1/70) (Pacific Blue)	Biolegend	101224
rIgG2b anti-mouse CD4 (Clone: GK1.5) (APC)	Biolegend	100412
rIgG2a anti-mouse CD8a (Clone: 53-6.7) (Pacific Blue)	Biolegend	100725
rIgG2a anti-mouse FOXP3 (Clone: FJK-16s) (PE)	Invitrogen	12-5773-82
mIgG2a anti-mouse CD45.2 (Clone: 104) (PE/Cy7)	Biolegend	109830
mIgG2a anti-mouse NK1.1 (Clone: PK136) (APC)	Biolegend	108710
rIgG2 c anti-mouse Ly-6C (Clone: HK1.4) (PerCP/Cy5.5)	Biolegend	128012
rIgG2a anti-mouse Ly-6G (Clone: 1A8) (APC/Cy7)	Biolegend	127624
rIgG2a anti-mouse CD5 (Clone: 53-7.3) (PerCP/Cy5.5)	Biolegend	100624
F(ab')$_2$ anti-mouse IgG (AlexaFluor 488)	Jackson ImmunoResearch	AB_2338861
Goat anti-human IgG (AlexaFluor 488)	Jackson ImmunoResearch	AB_2337831
hIgG1 anti-human hFcγRIIB (Clone: 6G11)	BioInvent International	https://doi.org/10.1016/j.ccell.2015.03.005
hIgG1 N297Q anti-human hFcγRIIB (Clone: 6G11)	BioInvent International	https://doi.org/10.1016/j.ccell.2015.03.005
hIgG1 N297Q anti-human hFcγRIIB (Clone: 6G08)	BioInvent International	https://doi.org/10.1016/j.ccell.2015.03.005
hIgG1 anti-human CD20 (Clone: Rituximab)	University Hospital Southampton Pharmacy	N/A
hIgG1 anti-human EGFR (Clone: Cetuximab)	University Hospital Southampton Pharmacy	N/A
mIgG1 anti-mouse CD20 (Clone: 18B12)	In-house	N/A
mIgG2a anti-mouse CD20 (Clone: 18B12)	In-house	N/A

(Continued on next page)

Continued		
REAGENT or RESOURCE	SOURCE	IDENTIFIER
mIgG1 anti-human CD20 (Clone: Rituximab)	In-house	N/A
mIgG2a anti-human CD20 (Clone: Rituximab)	In-house	N/A
rIgG1 anti-mouse CD25 (Clone: PC61)	In-house	N/A
rIgG1 anti-mouse CD79b (Clone: AT 107-2)	In-house	N/A
mIgG2a anti-mouse OX40 (Clone: OX86)	In-house	N/A
hIgG1 anti-human CD40 (Clone: chiLOB 7/4)	In-house	N/A
mIgG2a anti-human CD40 (Clone: chiLOB 7/4)	In-house	N/A
Rabbit anti-human CD32B (Clone: EP888Y)	Abcam	EP888Y
Rabbit anti-human CD32B (phospho Y292)	Abcam	EP926Y
anti-human/mouse SHIP1	Cell Signaling Technology	2728S
anti-human/mouse Phospho-SHIP1 (Tyr1020)	Cell Signaling Technology	3941S
anti-human/mouse alpha tubulin	Cell Signaling Technology	2144S
Biological samples		
Healthy human whole blood	Annoymised donor, University of Southampton	N/A
Chemicals, peptides, and recombinant proteins		
Ertholyse Red Cell Lysis Buffer	BioRad	BUF04B
OCT emedding matrix	Cell Path	KMA-0100-00A
VECTASHIELD® HardSet^{TM} Antifade Mounting Medium with DAPI	Vector Laboratories	H-1500-10
Alexa Fluor^{TM} 488 Antibody Labeling Kit	Invitrogen	A20181
Fluo-3, AM, Calcium Indicator	Invitrogen	F1242
Pluronic^{TM} F-127 (20% Solution in DMSO)	Invitrogen	P3000MP
Critical commercial assays		
B cell isolation kit, mouse	Miltenyi Biotec	130-090-862
Experimental models: Cell lines		
Mouse tumor: EG7	https://doi.org/10.1016/j.ccell.2020.04.013	N/A
Mouse tumor: Eμ-TCL1	https://doi.org/10.1038/leu.2016.333	N/A
πBCL1 cells	https://doi.org/10.1089/cbr.2000.15.581	N/A
Raji cells	ATCC	CCL-86
CHO-k1 cells	ATCC	CCL-61
Experimental models: Organisms/strains		
hCD20 Tg on C57BL/6J	https://doi.org/10.1182/blood-2008-04-149161	N/A
hCD40 Tg on C57BL/6J	https://doi.org/10.1016/j.ccell.2014.11.001	N/A
FcγRII$^{-/-}$ on C57BL/6J	https://doi.org/10.1182/blood-2008-04-149161	N/A
FcγRII$^{-/-}$ hFcγRIIB$^{-/+}$ on C57BL/6J	https://doi.org/10.1016/j.ccell.2015.03.005	N/A
FcγRII$^{-/-}$ NoTIM$^{-/+}$ on C57BL/6J	This paper	N/A
Oligonucleotides		
NoTIM mutation forward primer: AGGCTGACAAAGTTGGGGCTGAGAACACA ATCACCTTTTCACTTCTCATGCACCCGGATGC	This paper	N/A
NoTIM mutation reverse primer: GTTGCTGCTGTAGTGGCCTTGATCTTTTGCA GGAAAAAGCGGATTTCAGCCAATCCCACTAA TCCTGATG	This paper	N/A
Software and algorithms		
Prism	GraphPad	N/A
Flowjo	BD Biosciences	N/A
FCS Express	De Novo Software	N/A
Photoshop	Adobe	N/A

(*Continued on next page*)

Continued

REAGENT or RESOURCE	SOURCE	IDENTIFIER
SeqMan Pro	DNASTAR	N/A
PKSolver	https://doi.org/10.1016/j.cmpb.2010.01.007	N/A

RESOURCE AVAILABILITY

Lead contact

Further information and requests for resources and reagents should be directed to and will be fulfilled by the lead contact, Professor Mark Cragg (msc@soton.ac.uk).

Materials availability

All bespoke reagents generated in this study are available, where not constrained with third party agreements, from the lead contact with a completed Materials Transfer Agreement.

Data and code availability

- Data supporting the current study are available from the corresponding author upon request.
- This paper does not report original code.
- Any additional information required to reanalyze the data reported in this paper is available from the lead contact upon request.

EXPERIMENTAL MODEL AND SUBJECT DETAILS

Mice

Mouse (m) FcγRII$^{-/-}$ mice and human (h)CD20 Tg mice have been described previously (Beers et al., 2008). hCD40 Tg mice have been previously described (White et al., 2015). hFcγRIIB$^{-/+}$ mice have been described previously (Roghanian et al., 2015). For NoTIM$^{+/-}$ mice, the ITIM Y273F and Y254F mutation was generated using site-directed mutagenesis from the full length FCGR2B2 coding region amplified from the human Burkitt's lymphoma Raji cell cDNA and introduced into the mouse genome through microinjection of C57BL/6J oocytes by Cyagen. NoTIM$^{+/-}$ and hFcγRIIB$^{-/+}$ mice were intercrossed with mFcγRII$^{-/-}$ mice (C57BL/6J) to remove the endogenous mouse inhibitory receptor. NoTIM progeny were screened by PCR (amplifying genomic DNA extracted from ear tips) or FCM of peripheral blood. NoTIM$^{+/-}$ mFcγRII$^{-/-}$ mice were crossed with hCD20 transgenic (Tg) mice to generate hCD20^{+} x NoTIM$^{+/-}$ x mFcγRII$^{-/-}$ progeny. C57BL/6J, BALB/c and severe compromised immune deficiency (SCID) mice were purchased from Charles River and then bred and maintained in local animal facilities, alongside other strains, in accordance with the UK Home Office guidelines. NOD/SCID mice were purchased from Taconic (Bomholt, Denmark), and housed in local facilities in Lund, Sweden. All experiments were conducted under UK Home Office licenses PPL30/1269 and P4D9C89EA following approval by local ethical committees, reporting to the Home Office Animal Welfare Ethical Review Board (AWERB) at the University of Southampton and were carried out in accordance with the Animals (Scientific Procedures) Act 1986 and the GSK Policy on the Care, Welfare and Treatment of Animals or under Swedish Board of Agriculture guidelines with a general permit allowing animal work (31-11587/10). Experiments used both male and female mice and mice were age and sex matched within experiments. For the majority of experiments mice were aged between 16 and 24 weeks. Littermates of the same sex were randomly assigned to experimental groups at the start of the experiment. For the majority of experiments mice were maintained in SPF conditions in IVC caging. Food (irradiated RM1 (E)) and water was available ad libutum, mice were maintained on a 12-h light/dark cycle and environmental enrichment was provided; temperature was maintained between 20 and 24°C. Mice were visually checked daily if adverse effects were anticipated or if mice were nearing a humane endpoint.

Human samples

Human biological samples were sourced ethically with informed consent. Human B cells, T cells and monocytes were purified from human blood obtained from healthy donors with prior informed consent and Ethical approval from the East of Scotland Research Ethics Service, Tayside, UK. Donors were a mix of males and females under the age of 30. CLL samples were obtained through the Department of Hematology and Department of Oncology at Skånes University Hospital, Lund. Informed consent was provided in accordance with the Declaration of Helsinki. Ethical approval was obtained from the Ethics Committee of Skåne University Hospital.

Cell lines

All cell lines were maintained in a humidified incubator at 37°C and 5% CO_2. πBCL1 cells were grown in culture in RPMI supplemented with 2-mercaptoethanol (50 uM), glutamine (2 mM), pyruvate (1 mM), penicillin and streptomycin (100 IU/mL), amphotericin (2 mg/mL) and 20% fetal calf serum (FCS) (Illidge et al., 2000). EG7 and Raji cell lines were grown in RPMI supplemented with

10% FCS, 2 mM L-glutamine (2 mM), 1 mM pyruvate (1 mM), penicillin (100 IU/mL) and streptomycin (100 μg/mL). EG7 cells were cultured in the additional presence of 0.4 mg/mL geneticin.

METHOD DETAILS

Antibodies and reagents

Antibodies

Rituximab (hIgG1) and cetuximab (hIgG1) were kindly provided by University Hospital Southampton Pharmacy. 6G11 (hIgG1[BI-1206] (6G), hIgG1 N297Q mutant [BI-1607] (6G-Q)) and 6G08 (hIgG1) N297Q mutant were kindly provided by BioInvent International AB. 6G11 has been previously described (Roghanian et al., 2015). 18B12 (mIgG1, mIgG2a), PC61 (rIgG1), AT107-2 (rIgG1), OX86 (mIgG2a), chiLOB7/4 (mIgG2a, hIgG1) and Rituximab (mIgG1, mIgG2a) were produced in-house using stably transfected CHO-K1 cells. Purity was assessed by electrophoresis (Beckman EP; Beckman) and lack of aggregation confirmed by size exclusion (SEC) high performance liquid chromatography (HPLC). Unless otherwise stated, all antibodies were administered i.v. or i.p in 200 μL sterile PBS.

Flow cytometry (FCM)

Samples were stained with the appropriate antibody-fluorophore conjugate for 30 min at 4°C in the dark. Samples were then washed in ACK red cell lysis buffer or Erytholyse red blood cell lysing buffer (BioRad) and subsequently washed in FACS buffer (PBS, 1% BSA, 0.01% sodium azide). Antibodies used for staining can be found in Key reagents table. Samples were stored in the dark at 4°C until analysis. FACSCalibur and FACSCanto II (BD Biosciences) flow cytometers were used for data acquisition and results analyzed using FlowJo Version 10 (BD Biosciences).

Immunofluorescence

Tissues were frozen in OCT media (Cell Path) and placed in isopentane on a bed of dry ice. 10 μm frozen sections were then cut, fixed in acetone, and blocked with 5% normal goat serum before incubation with mAb to hFcγRIIB (EP888Y, Abcam), mFcγRII (AT130-5, in-house) or B cells (B220, BD Pharmingen) followed by goat anti-human-AF488 (Invitrogen), goat anti-rabbit-AF488 (Invitrogen) or goat anti-rat-AF647 (Invitrogen). Slides were mounted using Vectashield hardset with 4′,6-diamidino-2-phenylindole (DAPI; Vector Laboratories). Images were collected using a CKX41 inverted microscope using a Plan Achromat 10×0.25 objective lens (Olympus). RGB images (TIFF) were transferred to Adobe Photoshop CS6 and RGB image overlays created. Background autofluorescence was removed, contrast stretched, and brightness adjusted to maximize clarity, with all images treated equivalently.

Cell isolation

Mouse splenic B cells were purified by negative selection using a MACS B cell isolation kit (Miltenyi Biotec) and collected in supplemented RPMI (RPMI 1640 containing 2 mM glutamine, 1 mM pyruvate, 100 IU/mL penicillin and streptomycin and 10% FCS (Gibco).

Generation of mouse bone marrow-derived macrophages (BMDM)

Mouse BMDMs were generated from cells isolated from the femur and tibia of mice as previously reported (Williams et al., 2013). Briefly, bone marrow cells were cultured in supplemented RPMI containing 20% L929 cell-conditioned medium (in-house). Cells were cultured for 7 days at 37°C and 5% CO_2 before use. Macrophage differentiation was confirmed by morphology and F4/80 expression.

mCD20 mIgG1 internalization assay

Internalization assays were performed as detailed previously (Williams et al., 2013). In brief, isolated B cells were incubated with AF488-labelled anti-mCD20 mIgG1 (18B12) (5 μg/mL) at 37°C. At stated time points, cells were washed, resuspended, and incubated at 4°C for 30 min in the presence or absence of anti-AF488 quenching antibody (Invitrogen) before analysis via FCM. Results are presented as the percentage of internalized mAb (inversely proportional to the amount of surface accessible mAb) which was calculated as (unquenched MFI-quenched MFI)/unquenched × 100).

Calcium flux assay

Calcium mobilization was measured using the fluorescent probe Fluo-3-AM. Isolated splenic B cells at 1 × 10^7 cells/mL were incubated with 10 μ M of Fluo-3-AM (Invitrogen) and 0.002% (v/v) Pluronic F-127 (Invitrogen) for 30 min at 37°C. Cells were then washed and resuspended in supplemented RPMI media for 15 min at 37°C. Cells were then incubated with 10 μg/mL 6G08 (hIgG1 N297Q mutation) or isotype controls for another 15 min at 37°C. Cells were kept warm prior to data acquisition of background fluorescence, followed by the addition of 20 μg/mL goat $F(ab')_2$ anti-mouse IgM (Jackson). Acquisition was continued for 2.5 min before the addition of 0.6 μ M ionomycin to elicit robust calcium flux.

Preparation of heat aggregated human IgG

Human IgG was treated at 62°C for 30 min to induce aggregation. The heat aggregated IgG was then separated from the monomeric fraction by size exclusion HPLC.

Heat aggregated human IgG internalization assay

Heat-aggregated-human IgG internalization assay was performed as previously described (Vaughan et al., 2015). In brief, isolated splenic B cells at 1 × 10^6 cells/mL were treated with 20 μg/mL for 30 min at 4°C. Cells were then washed and divided into three fractions. One fraction was maintained at 4°C for 60 min (time zero), another fraction was maintained at 37°C for 30 min and 4°C for 30 min (time 30 min) and the last fraction was maintained at 37°C for 60 min (time 60 min). All fractions were then stained with AF488 labelled polyclonal goat anti-human IgG (Jackson) and the MFI was quantified using FCM. Internalization was expressed as the proportion of ahIgG remaining at the cell surface compared to time zero using the following formula: % cell surface ahIgG = (MFI of internalized fraction/MFI of time zero fraction) x 100.

Western Blotting

To assess the activity of the ITIM signaling pathway, 2–5x10^6 cells isolated splenic B cells or BMDMs were washed in RPMI and cultured in supplemented RPMI before addition of irrelevant, hFcγRIIB agonist (6G08) or hFcγRIIB antagonist (6G-Q) mAb (10 μg/mL) in hIgG1 N297Q formats at 37°C for either 30 min (B cells) or 15 min (BMDMs). Cells were subsequently washed with cold PBS and lysed in Onyx buffer (containing a cocktail of protease and phosphatase inhibitors). Samples were separated by SDS PAGE on 8–10% Bis-Tris gels (Invitrogen) in MOPS running buffer. Gels were transferred to a PDVF membrane using the iBlot 2 Transfer System (Invitrogen) and blocked in 5% BSA-TBS-T for 1 h. The membrane was then incubated with primary antibodies for 16 h at 4°C, washed and then incubated with HRP-conjugated secondary IgG (Sigma Aldrich) for 1.5 h at RT. ECL substrate (GE Healthcare) was used for detection and samples were imaged using the Chemi Doc-it imaging system (UVP). Antibodies against hFcγRIIB (EP888Y, Abcam) phosphorylated hFcγRIIB (EP926Y, Abcam), SHIP-1 (2728, Cell Signaling Technology), phosphorylated SHIP1 (3941, Cell Signaling Technology) and alpha-tubulin (2144, Cell Signaling Technology) were used for Western Blotting.

Cell binding assay

Serum anti-mCD20 mIgG1 (18B12) titers were determined by a πBCL1 cell binding assay. Serially diluted serum samples were incubated with 0.5 × 10^6 cells/mL for 15 min at room temperature in supplemented RPMI. Cells were then washed and stained using an anti-mouse Fc-FITC secondary antibody (Jackson) for 30 min at 4°C. Cells were then washed and analyzed by FCM. A standard curve was generated from a known concentration of anti-mCD20 mIgG1 and used to determine anti-mCD20 IgG serum concentrations.

Adoptive B cell transfer assay

The relative depletion of adoptively transferred target B cells was performed as detailed previously (Beers et al., 2010). In brief, 2 × 10^7 splenocytes/mL were stained as target or non-target cells with 5 μM or 0.5 μM CFSE, respectively. Labelled cells were then quenched using an equal volume of FCS and washed before being combined in a 1:1 ratio and injected i.v into recipient mice (~5–10 × 10^6 cells/mouse) on Day 0. Mice were then treated with a hFcγRIIB blocking antibody (6G, 6G-Q or isotype) i.p., and i.v. with 2 mg/kg rituximab (RTX) (or isotype) or 5 mg/kg hCD40 antibody (chiLOB7/4) according to the experimental schedule. Mice were then culled 16 h following treatment with rituximab to examine the percentage of CFSE positive B cells in the blood and spleen and bone marrow using FCM. Further staining of FcγRs on relevant immune effector cells was carried out at the same time.

In vivo Raji xenograft therapy

2.5 × 10^6 Raji lymphoma cells were injected (i.v.) into SCID mice. 1 week later mice were treated with 5 mg/kg of either RTX, 6G-Q or a combination of both and thereafter on a weekly basis up to 4 times. Animals were monitored over time and sacrificed upon evidence of terminal tumor development according to experimental end-points with % survival calculated.

CLL patient derived xenograft model

CLL patient PBMCs were isolated and injected (6–10 × 10^7 cells) i.v. into irradiated (1 Gy) NOD/SCID immunocompromised mice, as detailed previously (Roghanian et al., 2015). Four to five days later, mice were treated with 1–10 mg/kg of hCD20 mAb (RTX), hFcγRIIB blocking mAb (6G-Q), or both mAbs (i.p.), with a second injection 2–3 days later. Mice were sacrificed 2–3 days after the final injection, spleens were harvested, and human cells identified and quantified as CD45+ CD5+ CD19+ using FCM.

In vivo B cell depletion

Depletion of B cells in response to escalating concentrations of the anti-mCD20 mAb 18B12 was determined using FCM. In brief, tail blood was collected prior to any mAb treatment and stained using fluorescent antibodies against mouse CD19 and B220. The percentage of $CD19^+B220^+$ cells as a percentage of lymphocytes was established and normalized to 100%. Following mAb treatment, mice were tail bled according to the experimental schedule and B cells were identified as above. The percentage of $CD19^+B220^+$ cells as a percentage of lymphocytes was then normalized to the pre-bleed to ascertain the percentage of B cells remaining.

In vivo regulatory T cell depletion using PC61

Mice were treated with 250 μg of PC61 or AT107-2 (both rIgG1) i.p. on Day 0. Mice were then tail bled according to the experimental schedule to establish the percentage of peripheral regulatory T cells using FCM. In brief, tail blood was stained with fluorescent

antibodies against mouse CD4, CD8 and the intracellular transcription factor FOXP3. Treg cells were identified as $CD8^- CD4^+ FOXP3^+$ and were expressed as a percentage of $CD8^- CD4^+$ cells. On the final day mice were culled and Treg cells were analyzed in the blood and spleen.

In vivo regulatory T cell depletion using OX86 in E.G7 tumor bearing mice

5×10^5 E.G7 tumors cells in 100 μL PBS were injected S.C. into the right-hand flank of mice. Tumors were measured using electronic calipers (Draper). Once tumors were established (5×5 - 7×7 mm^2) mice were treated with 2 × 200 ug shots of antibody (anti-mOX40 (OX86) mIgG2a or anti-hCD20 (rituximab) mIgG2a as an isotype control) on Day 0 and Day 2 via i.p injection. Mice were then bled on Day 2 and Day 4 to ascertain Treg depletion within the periphery. Briefly, blood was analyzed by FCM and based on $CD4^+$, $CD8^+$ and $FOXP3^+$ expression. T cell populations were enumerated using Precision Counting Beads (Biolegend) according to the manufacturer's instructions. Mice kept for long term survival were also bled on Day 9. Tumor size was monitored 3 times a week until experimental endpoint was reached as determined by a tumor size of 15×15 mm^2.

In vivo Eμ-TCL1 lymphoma depletion

The depletion of Eμ-TCL1 cells *in vivo* was performed as previously described (Carter et al., 2017). In brief, mice were given 5×10^6 Eμ-TCL1 cells via i.p. injection. Tumor load was monitored every 7 days by assessing the percentage of Eμ-TCL1 cells in peripheral blood. In brief, mice were tail bled and were assessed for the percentage of $CD19^+CD5^+B220^{lo}$ cells as a percentage of total lymphocytes by FCM. When tumor load reached 10–20% of lymphocytes, mice were treated with 100 μg antibody (anti-mCD20 mIgG2a (18B12) or anti-hCD20 (rituximab) mIgG2a as an isotype control) via i.p. injection. Mice were then bled on Day 2 and Day 7 to monitor tumor load. Mice were bled once a week from treatment until experimental endpoint was reached which was defined as two of the three following criteria being met: Eμ-TCL1 cells as a percentage of lymphocytes exceeding 80%, a white blood cell count of $>5 \times 10^7$ cells/mL and a splenomegaly score of 3 or above (approximately 3 cm long). Eμ-TCL1 cells were monitored using FCM, the white blood cell count was also monitored by FCM using Precision Count Beads (Biolegend) according to the manufacturer's instructions.

QUANTIFICATION AND STATISTICAL ANALYSIS

FCM data analysis was performed using either FCS Express software Version 3 (De Novo Software) or Flowjo Version 10.6 (BD Biosciences). All other data analysis were performed using GraphPad Prism versions 7–9 (GraphPad Software). Pharmacokinetic analysis was carried out using the PKSolver tool (Zhang et al., 2010). Statistical significance between two factors was analyzed using a two-tailed unpaired t-test. Statistical significance between groups was assessed by using a one-way ANOVA test unless otherwise stated. Multiple comparison tests were used as appropriate and are detailed in figure legends. The Shapiro–Wilk test was used to test for normality and determine the use of parametric or non-parametric analyses. The statistical significance in long term survival experiments was analyzed using Kaplan-Meier survival curve with the Mantel-Cox test used to assess significance between groups. Throughout, $*p < 0.05$, $**p < 0.01$, $***p < 0.001$, $****p \leq 0.0001$.

Cell Reports, Volume *40*

Supplemental information

FcγRIIB controls antibody-mediated target cell depletion by ITIM-independent mechanisms

Alexander P. Simpson, Ali Roghanian, Robert J. Oldham, H.T. Claude Chan, Christine A. Penfold, Hyung J. Kim, Tatyana Inzhelevskaya, C. Ian Mockridge, Kerry L. Cox, Yury D. Bogdanov, Sonya James, Alison L. Tutt, Daniel Rycroft, Peter Morley, Lekh N. Dahal, Ingrid Teige, Björn Frendeus, Stephen A. Beers, and Mark S. Cragg

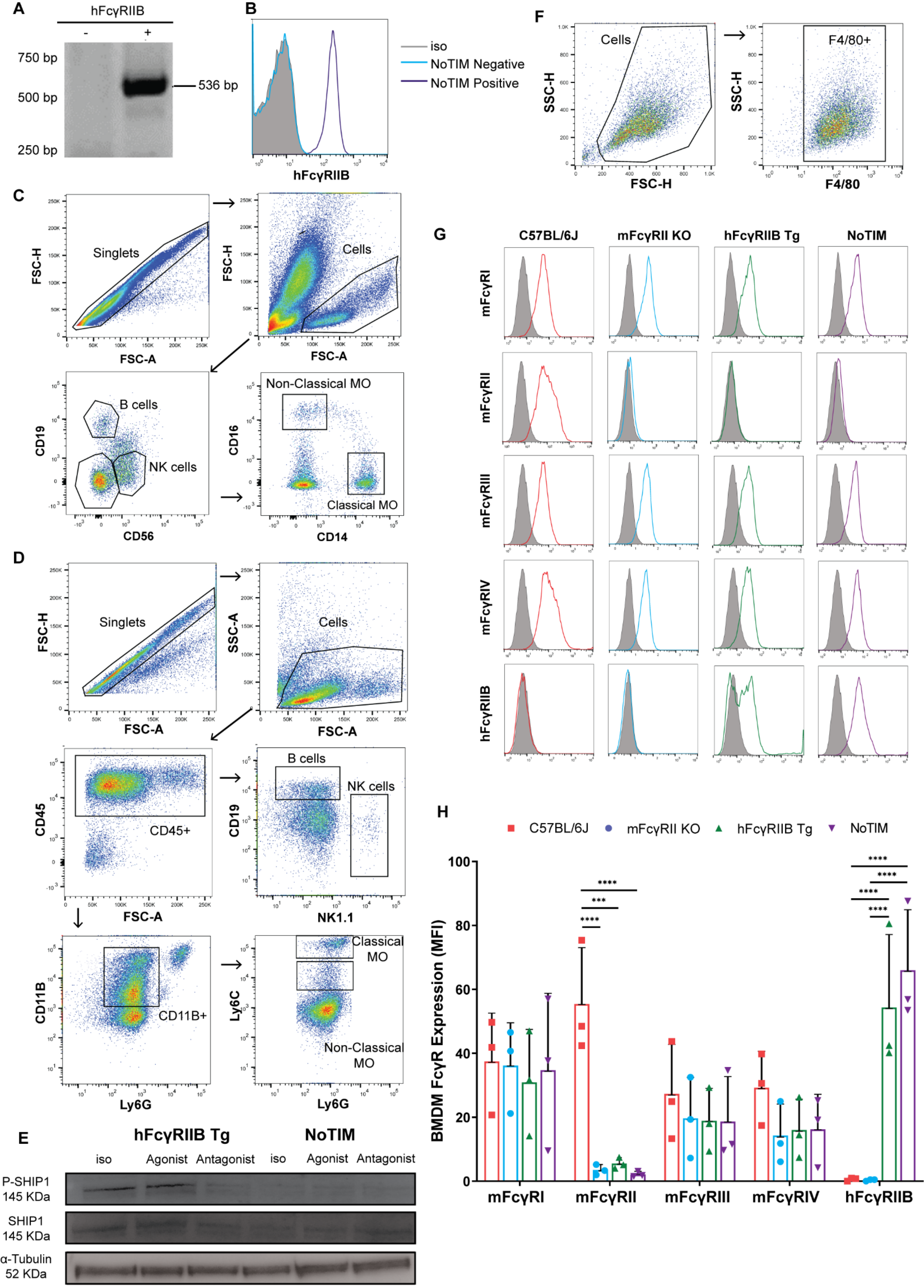
A
hFcγRIIB
-
+
750 bp
500 bp
250 bp
536 bp
B
iso
NoTIM Negative
NoTIM Positive
hFcγRIIB
C
FSC-H
FSC-A
Singlets
Cells
CD19
CD56
B cells
NK cells
Non-Classical MO
Classical MO
CD16
CD14
D
SSC-A
CD45
CD45+
NK1.1
CD11B
Ly6G
CD11B+
Ly6C
Classical MO
Non-Classical MO
E
hFcγRIIB Tg
NoTIM
iso
Agonist
Antagonist
P-SHIP1 145 KDa
SHIP1 145 KDa
α-Tubulin 52 KDa
F
SSC-H
FSC-H
F4/80
F4/80+
G
C57BL/6J
mFcγRII KO
hFcγRIIB Tg
NoTIM
mFcγRI
mFcγRII
mFcγRIII
mFcγRIV
hFcγRIIB
H
BMDM FcγR Expression (MFI)
****
***
100
80
60
40
20
0

FIGURE S1

Figure S1, related to Figure 2. Characterization of the hFcγRIIB NoTIM mouse. A) The presence of the NoTIM transgene was assessed by PCR amplification from genomic DNA using gene-specific primers to identify positive and negative progeny. The transgene included the 400bp region upstream of the human start codon to foster human tissue-specific gene expression. B) flow cytometry was then used to confirm the cell surface expression of the receptor. Grey: isotype; blue: negative sample; purple: NoTIM sample. Representative histogram gated on $B220^{+}CD19^{+}$ cells. C) Flow cytometric gating strategy used to identify human B cells, NK cells, classical and non-classical monocytes (MO) as used in Fig. 2C. D) Flow cytometric gating strategy used to identify murine B cells, NK cells, classical and non-classical monocytes (MO) as used in Fig. 2C. E) Splenic B cells were isolated from NoTIM and hFcγRIIB Tg mice and stimulated with hFcγRIIB specific mAb. Lysates were then probed for phosphorylated SHIP-1 (P-SHIP1), total SHIP-1 and α tubulin as a loading control. F-H) To understand if the loss of the endogenous mFcγRII and the introduction of the hFcγRIIB transgene impacted on the expression of activating mFcγRs, BMDMs were differentiated from the various mouse strains and flow cytometry used to investigate cell surface expression of FcγRs. Expression of FcγR on BMDMs derived from C57BL/6J WT, mFcγRII $^{-/-}$, hFcγRIIB Tg and NoTIM mice is shown. Gating shown in (E) with representative histograms for FcγR shown in (F) and the mean fluorescence intensity (MFI) from 3 independent experiments combined in (G); grey = isotype control. Color represents the relevant FcγR. Columns represent the mean (+SD). Statistical analyses were conducted using a one-way ANOVA with Tukey's multiple comparisons test. * $P \leq 0.05$, ** $P \leq 0.01$, *** $P \leq 0.001$, **** $P \leq 0.0001$.

FIGURE S2

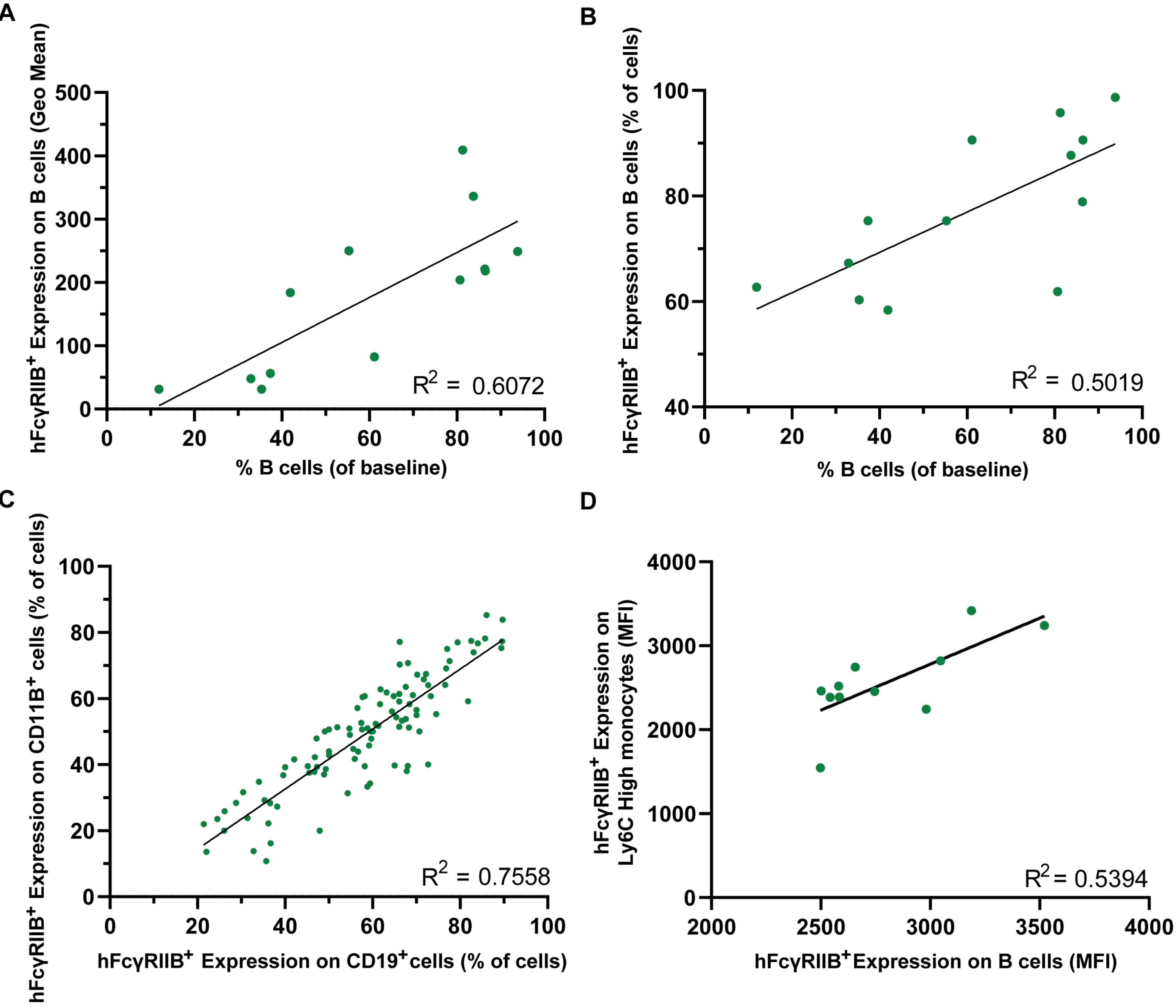
A
hFcγRIIB+ Expression on B cells (Geo Mean)
% B cells (of baseline)
R2 = 0.6072
B
hFcγRIIB+ Expression on B cells (% of cells)
% B cells (of baseline)
R2 = 0.5019
C
hFcγRIIB+ Expression on CD11B+ cells (% of cells)
hFcγRIIB+ Expression on CD19+ cells (% of cells)
R2 = 0.7558
D
hFcγRIIB+ Expression on Ly6C High monocytes (MFI)
hFcγRIIB+ Expression on B cells (MFI)
R2 = 0.5394

Figure S2, related to Figure 3. Relationship between hFcγRIIB Tg expression and anti-mCD20 mAb-mediated depletion. A) The expression of hFcγRIIB on B cells from hFcγRIIB Tg mice was measured by flow cytometry prior to the anti-mCD20 (α-mCD20) mAb treatment and correlated to the % remaining B cells (as a % of pre-bleed) on day 14 after treatment from experiments shown in Fig. 4. The R^2 values were calculated for from the line of best fit (R^2 = 0.6072; p 0.0017). B) The % of hFcγRIIB positive B cells from hFcγRIIB Tg mice was measured by flow cytometry prior to α-mCD20 mAb treatment and correlated to the % remaining B cells (as a % of pre-bleed) on day 14 from experiments shown in Fig. 4 (R^2 = 0.5019; p 0.0067). C) Correlation of % positivity of the hFcγRIIB transgene between B cells and monocytes (R^2 0.7558; p <0.0001). Given the tight correlation, and ease of screening the majority of mice were screened solely for hFcγRIIB expression on B cells. D) The MFI of hFcγRIIB on splenic Ly6C high monocytes from healthy hFcγRIIB Tg mice was measured by flow cytometry and correlated with the MFI of hFcγRIIB on splenic B cells. The R^2 values were calculated for from the line of best fit (R^2 = 0.5394; p 0.0101).

FIGURE S3

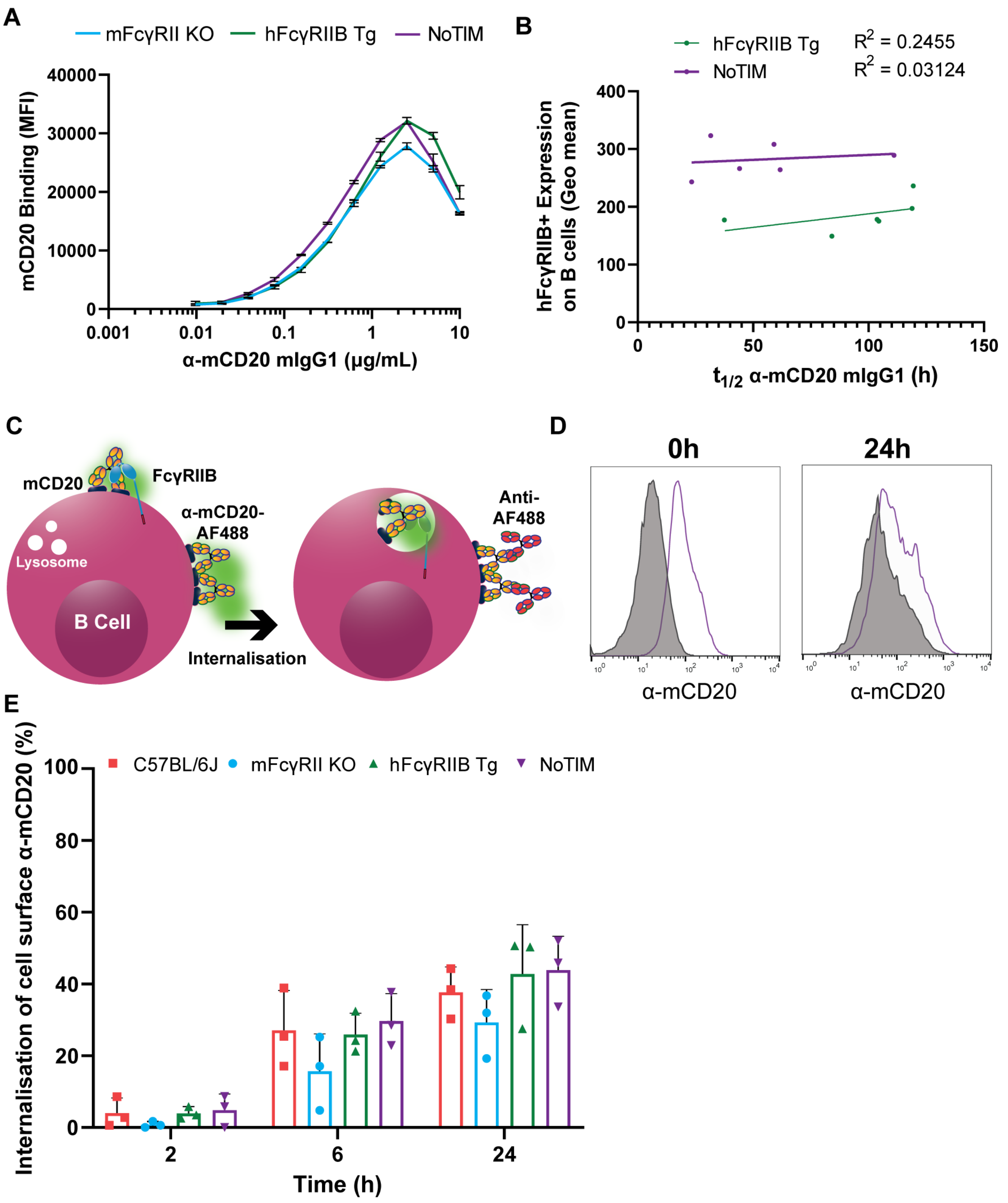
A
mFcγRII KO
hFcγRIIB Tg
NoTIM
mCD20 Binding (MFI)
40000
30000
20000
10000
0
0.001
0.01
0.1
1
10
α-mCD20 mIgG1 (μg/mL)
B
hFcγRIIB Tg
NoTIM
R2 = 0.2455
R2 = 0.03124
hFcγRIIB+ Expression on B cells (Geo mean)
400
300
200
100
0
0
50
100
150
t1/2 α-mCD20 mIgG1 (h)
C
mCD20
FcγRIIB
α-mCD20-AF488
Lysosome
B Cell
Internalisation
Anti-AF488
D
0h
24h
α-mCD20
α-mCD20
E
C57BL/6J
mFcγRII KO
hFcγRIIB Tg
NoTIM
Internalisation of cell surface α-mCD20 (%)
100
80
60
40
20
0
2
6
24
Time (h)

Figure S3, related to Figure 4. Impairment of B cell depletion in NoTIM mice is not due to insufficient serum levels or rapid internalization of mAb. A) Titration and saturation binding curve of anti-mCD20 (α-mCD20) mIgG1 binding to mouse B cells from mFcγRII$^{-/-}$, hFcγRIIB Tg and NoTIM mice; indicating a saturation level of ~2.5 µg/ml in each case. B) Relationship between hFcγRIIB Tg expression and $t_{1/2}$ in hFcγRIIB Tg and NoTIM mice. No clear correlation was observed, indicating that FcγRIIB expression does not influence α-mCD20 mAb serum levels. C, D and E) Splenic B cells were isolated from WT, mFcγRII$^{-/-}$, hFcγRIIB Tg and NoTIM mice and incubated in vitro with anti-mCD20-Alexa 488 mIgG1 for 2, 6 or 24 hours. Cells were then washed and then incubated in the presence or absence of anti-Alexa 488 quenching mAb as indicated in the schematic (C). D) indicates representative data after 0 and 24h. The fluorescence remaining after quenching indicates the proportion of internalized mAb, with % internalization calculated, and results plotted from 3 independent experiments in E) (conducted with triplicate samples). Columns represent means (+SD). Red: C57BL/6J WT; mFcγRII$^{-/-}$: blue; hFcγRIIB Tg: green; and NoTIM: purple. The data show that the presence of any FcγRIIB (mouse or human, WT or NoTIM) increases internalization but that this does not differ between the different FcγRIIB molecules.

FIGURE S4

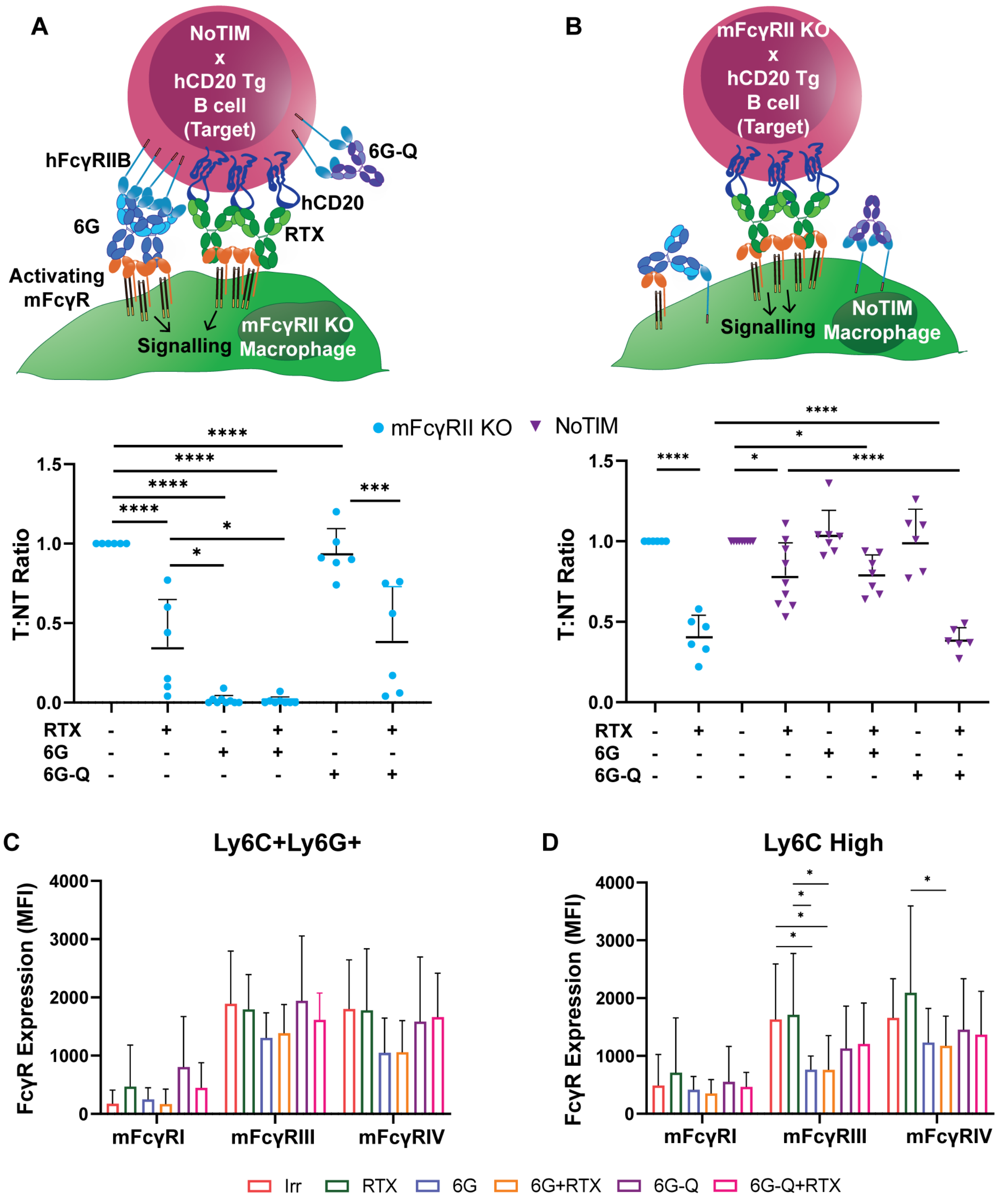
A
NoTIM
x
hCD20 Tg
B cell
(Target)
hFcγRIIB
6G-Q
hCD20
6G
RTX
Activating
mFcγR
mFcγRII KO
Macrophage
Signalling
B
mFcγRII KO
x
hCD20 Tg
B cell
(Target)
Signalling
NoTIM
Macrophage
mFcγRII KO
NoTIM
T:NT Ratio
1.5
1.0
0.5
0.0
****
***
*
RTX - + - + - +
6G - - + + - -
6G-Q - - - - + +
T:NT Ratio
RTX - + - + - + - +
6G - - - - + + - -
6G-Q - - - - - - + +
C
Ly6C+Ly6G+
FcγR Expression (MFI)
4000
3000
2000
1000
0
mFcγRI
mFcγRIII
mFcγRIV
D
Ly6C High
FcγR Expression (MFI)
Irr
RTX
6G
6G+RTX
6G-Q
6G-Q+RTX

Figure S4, related to Figure 5. NoTIM hFcγRIIB impairs depletion through expression on effector cells not target cells. A) NoTIM hFcγRIIB^{+} hCD20^{+} mFcγRII$^{-/-}$ target (T) and NoTIM hFcγRIIB^{+} mFcγRII$^{-/-}$ hCD20^{-} non-target (NT) splenocytes were injected i.v. into mFcγRII$^{-/-}$ mice (as detailed in Fig. 5). Mice were treated as indicated and on day 2, spleens were taken and assessed for the expression of FcγRs at the surface of F4/80^{+} myeloid effector cells. The upper panel represents the schema, with data representing depletion of target cells within the bone marrow showing mean (+SD) from 2 independent experiments below. Statistical analyses were conducted using a one-way ANOVA with Tukey's multiple comparisons test. B) mFcγRII$^{-/-}$ x CD20 Tg target (T) and mFcγRII$^{-/-}$ non-target (NT) splenocytes were injected into NoTIM mice. Mice were treated as indicated and on day 3, spleens were taken and assessed for the expression of FcγRs at the surface of myeloid cells. Above represents the experimental schema with the depletion of target cells within the bone marrow below. Statistical analyses were conducted using a one-way ANOVA with Sidak's multiple comparisons test. C) represents summed expression of FcγRs on splenic Ly6C+Ly6G+ cells. D) is the summed data of expression on Ly6C High cells. Bar = mean (+SD) from 2-3 independent experiments. Statistical analyses were conducted using a one-way ANOVA with Tukey's multiple comparisons test. * $P \leq 0.05$, *** $P \leq 0.001$, **** $P \leq 0.0001$. In summary detection of mFcγRIII and IV on both Ly6G+Ly6C+ and Ly6C^{hi} cells was reduced by the presence of 6G (in the presence or absence of RTX) but not by 6G-Q (in the presence or absence of RTX) when the NoTIM is expressed only on the effector cells and not the targets.

FIGURE S5

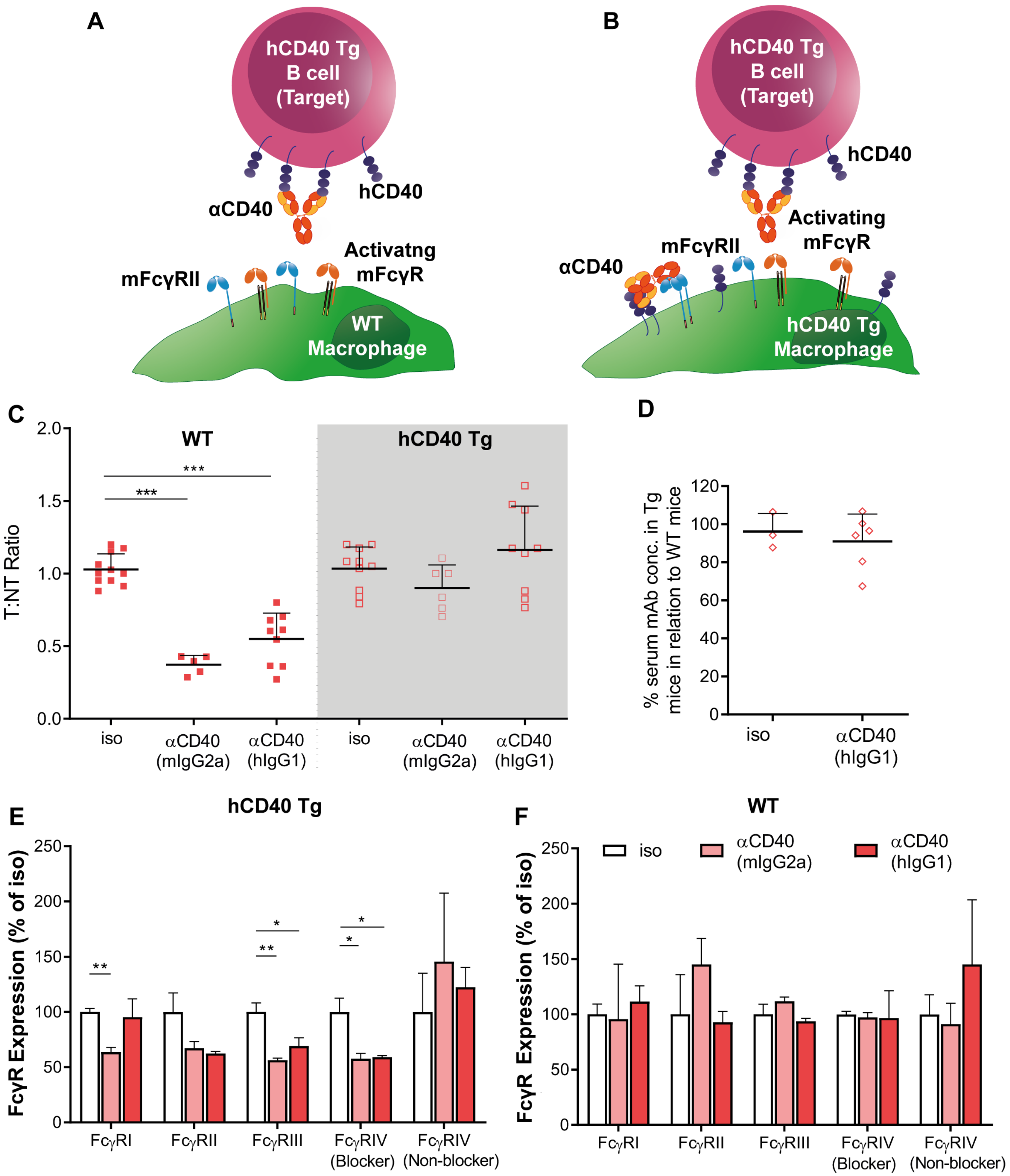
A
hCD40 Tg
B cell
(Target)
αCD40
hCD40
Activatng
mFcγR
mFcγRII
WT
Macrophage
B
hCD40 Tg
B cell
(Target)
hCD40
Activating
mFcγR
mFcγRII
αCD40
hCD40 Tg
Macrophage
C
WT
hCD40 Tg
T:NT Ratio
***
***
iso
αCD40
(mIgG2a)
αCD40
(hIgG1)
D
% serum mAb conc. in Tg
mice in relation to WT mice
iso
αCD40
(hIgG1)
E
hCD40 Tg
FcγR Expression (% of iso)
**
*
**
*
*
FcγRI
FcγRII
FcγRIII
FcγRIV
(Blocker)
FcγRIV
(Non-blocker)
F
WT
iso
αCD40
(mIgG2a)
αCD40
(hIgG1)

Figure S5, related to Figure 5. Concurrent expression of hCD40 on target and effector cells limits target cell depletion. (A, B) hCD40 Tg (T) and WT (NT) splenocytes were injected into WT (as indicated in panel A) or hCD40 Tg recipient mice (as indicated in panel B) on day 0. Next day, mice received anti-human CD40 (αCD40) mAb, mIgG2a or hIgG1 and the following day the ratio of splenic $CD19^{+}$ cells were determined, as before (C; data combined from 2-4 independent experiments, each point represents a single mouse (n = 6-10 mice/group). Results in WT mice indicated by filled symbols; left unshaded panel or in hCD40 Tg mice by open symbols; right grey panel. D) Serum was collected 1 day after injection of αCD40 mAb (hIgG1) and quantified by ELISA (n = 3-6 mice/group). E-F) Expression of indicated activating and inhibitory FcγRs was assessed on splenic F4/80+ macrophages in (E) hCD40 Tg and (F) WT mice (n = 5 mice/group). Bars represent mean (+SD). * $P \leq 0.05$, ** $P \leq 0.01$.

FIGURE S6

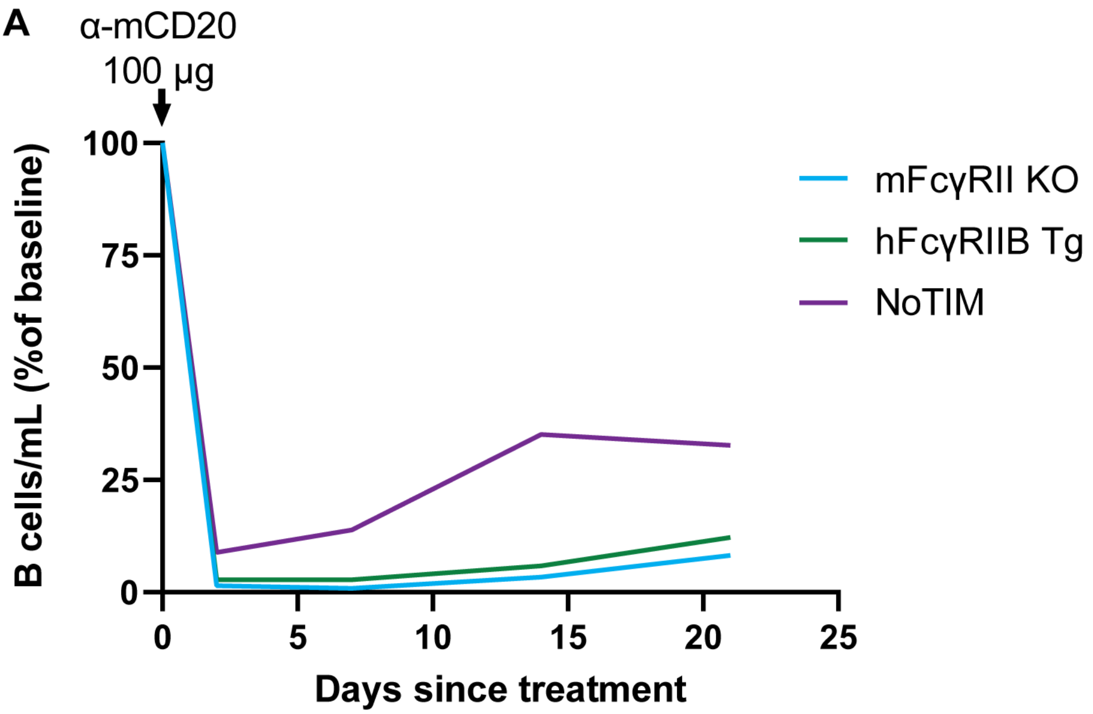
A
α-mCD20
100 µg
B cells/mL (%of baseline)
100
75
50
25
0
0
5
10
15
20
25
Days since treatment
mFcγRII KO
hFcγRIIB Tg
NoTIM

Figure S6, related to Figure 7. NoTIM hFcγRIIB impairs depletion of B cells in the Eu-TCL1 model. mFcγRII$^{-/-}$, hFcγRIIB Tg or NoTIM mice were injected with $5x10^6$ Eμ-TCL1 cells and monitored for tumor growth. When tumor equated to 10-20% of peripheral lymphocytes, mice were treated with 100 µg anti-mCD20 (α-mCD20) mIgG2a or isotype control. Flow cytometry was used to assess depletion of both normal B cells (CD19$^+$ B200^{hi} CD5$^-$); plotted is the kinetics of B cell depletion in each mouse model following treatment with mAb. Line = mean. The result of 3 independent experiments (n = 8-9 mice per group), The data shows that normal B cells are effectively deleted from the periphery in the presence of the Eμ-TCL1 cells at day 2 in all mice but that the B cells in the NoTIM mice recover more rapidly back to normal levels compared to mFcγRII$^{-/-}$ mice with hFcγRIIB Tg mice intermediate.
